# Inflammatory Response and Defects on Myelin Integrity in the Olfactory System of K18hACE2 Mice Infected with SARS-CoV-2

**DOI:** 10.1523/ENEURO.0106-24.2024

**Published:** 2024-06-12

**Authors:** Eduardo Martin-Lopez, Bowen Brennan, Tianyang Mao, Natalie Spence, Sarah J. Meller, Kimberly Han, Nawal Yahiaoui, Chelsea Wang, Akiko Iwasaki, Charles A. Greer

**Affiliations:** ^1^Department of Neurosurgery, Yale University School of Medicine, New Haven, Connecticut 06520-8082; ^2^Department of Neuroscience, Yale University School of Medicine, New Haven, Connecticut 06520-8001; ^3^Department of Immunobiology, Yale University School of Medicine, The Anlyan Center, New Haven, Connecticut 06520-8043; ^4^Yale University School of Public Health, New Haven, Connecticut 06520-0834; ^5^Howard Hughes Medical Institute, Chevy Chase, Maryland 20815; ^6^Interdepartmental Neuroscience Program, Yale University School of Medicine, New Haven, Connecticut 06520-8074

**Keywords:** inflammation, microglia, olfactory bulb, olfactory system, piriform cortex, SARS-CoV-2

## Abstract

Viruses, such as severe acute respiratory syndrome coronavirus 2 (SARS-CoV-2), use respiratory epithelial cells as an entry point for infection. Within the nasal cavity, the olfactory epithelium (OE) is particularly sensitive to infections which may lead to olfactory dysfunction. In patients suffering from coronavirus disease 2019, deficits in olfaction have been characterized as a distinctive symptom. Here, we used the K18hACE2 mice to study the spread of SARS-CoV-2 infection and inflammation in the olfactory system (OS) after 7 d of infection. In the OE, we found that SARS-CoV-2 selectively targeted the supporting/sustentacular cells (SCs) and macrophages from the lamina propria. In the brain, SARS-CoV-2 infected some microglial cells in the olfactory bulb (OB), and there was a widespread infection of projection neurons in the OB, piriform cortex (PC), and tubular striatum (TuS). Inflammation, indicated by both elevated numbers and morphologically activated IBA1^+^ cells (monocyte/macrophage lineages), was preferentially increased in the OE septum, while it was homogeneously distributed throughout the layers of the OB, PC, and TuS. Myelinated OS axonal tracts, the lateral olfactory tract, and the anterior commissure, exhibited decreased levels of 2′,3′-cyclic-nucleotide 3′-phosphodiesterase, indicative of myelin defects. Collectively, our work supports the hypothesis that SARS-CoV-2 infected SC and macrophages in the OE and, centrally, microglia and subpopulations of OS neurons. The observed inflammation throughout the OS areas and central myelin defects may account for the long-lasting olfactory deficit.

## Significance Statement

Damage to the olfactory system that can lead to loss of olfaction during coronavirus disease 2019 remains controversial. Using the K18hACE2 mouse infected with SARS-CoV-2, we show the infection of sustentacular cells and lamina propria macrophages in the olfactory epithelium but not the olfactory sensory neurons. In the brain, we found a widespread infection of projection neurons in the olfactory bulb (OB), piriform cortex, and tubular striatum, with microgliosis. Some SARS-CoV-2–infected microglia were observed in the OB. Alterations to the integrity of myelin in the olfactory tracts were evident. These data support the hypothesis of a nonolfactory entry pathway for SARS-CoV-2 into the brain, as well as the presence of impairments in neuronal conductivity in the olfactory tracts.

## Introduction

**Figure 1.**
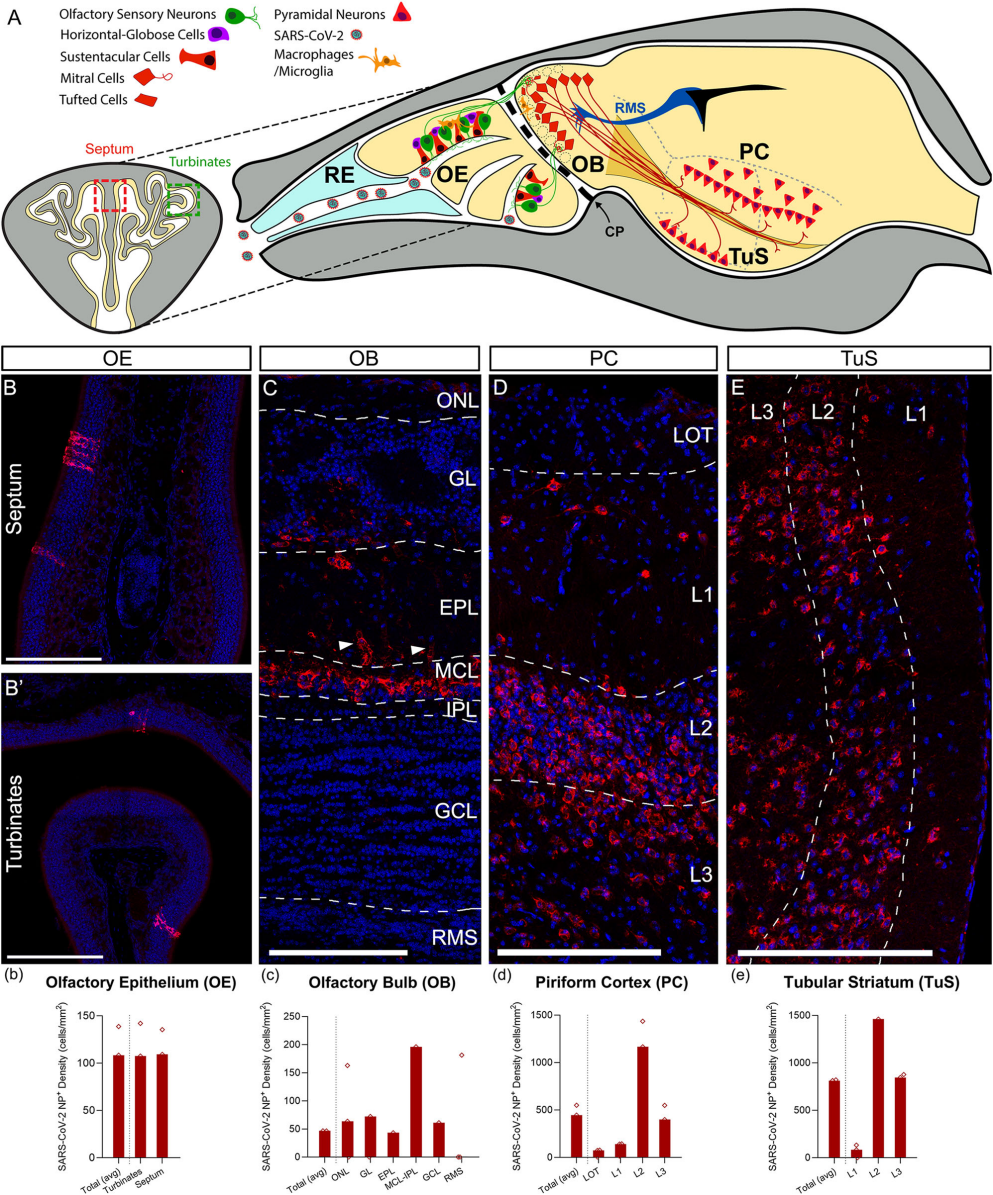
SARS-CoV-2 infection across the OS. ***A***, Diagram illustrating a coronal (OE) and sagittal section (entire OS and brain) of K18hACE2 mice. Cells infected by SARS-CoV-2 are shown in red (neuronal) and orange (monocyte–macrophage lineages). ***B***–***E***, Detection of SARS-CoV-2 nucleocapsid (red) with nuclei counterstained with DAPI (blue). ***B***–***b***, SARS-CoV-2–infected cells appear as small clusters in the septum (***B***) and turbinates (***B*’**). Quantification shows similar numbers of infected cells in both regions (***b***). ***C***, ***c***, SARS-CoV-2^+^ cells in the OB are predominantly accumulated around the MCL-IPL. Lower numbers are found in all other layers of the OB, including the most internal and external parts of the GL and EPL, respectively (***C***). Labeling of blood vessels is found in the EPL (***C***, arrowheads). ***D***, ***d***, SARS-CoV-2^+^ cells in PC showing extensive staining in the densely packed cell Layers 2 and 3 and in horizontal cells in Layer 1, located beneath the LOT (***D***). ***E***, ***e***, SARS-CoV-2^+^ cells in TuS accumulated mostly in the densely packed cell Layers 2 and 3. CP, cribriform plate; EPL, external plexiform layer; GCL, granule cell layer; GL, glomerular layer; IPL, internal plexiform layer; LOT, lateral olfactory tract; MCL, mitral cell layer; OB, olfactory bulb; OE, olfactory epithelium; ONL, olfactory nerve layer; PC, piriform cortex; RE, respiratory epithelium; RMS, rostral migratory stream (bulbar part); TuS, tubular striatum. Statistics in Table 2. Scale bars: 200 µm.

In 2019, SARS-CoV-2 (severe acute respiratory syndrome coronavirus 2) was identified as the pathological agent underlying coronavirus disease 2019 (COVID-19; Bergmann and Silverman, 2020; Huang et al., 2020). SARS-CoV-2 is characterized in part by the rapid infection of cells in the nasal cavity. The virus easily spreads into the lungs and other organs, causing severe inflammatory reactions and tissue damage (Brodin, 2021; Lamers and Haagmans, 2022; Merad et al., 2022; Priyal et al., 2023). The nasal cavity mucosa is covered by two epithelia: a ciliated, nonolfactory epithelium or respiratory epithelium (RE) that moistens and protects the airway from pathogens and the olfactory epithelium (OE) that contains the olfactory sensory neurons (OSNs; Fig. 1*A*; Harkema et al., 2006). In addition, OE includes sustentacular cells (SCs) or supporting cells that surround the OSNs. RE and SC express the angiotensin-converting enzyme 2 (ACE2) which is used for cell entry by SARS-CoV-2 and the protease TMPRSS2 which is important for cleavage and priming of the spike protein (Bilinska et al., 2020; Cooper et al., 2020; Fodoulian et al., 2020; Lee et al., 2020; Blume et al., 2021). Initially, the infection of SCs can impact the OSNs by limiting their nutrient support (Bilinska and Butowt, 2020; Khan et al., 2021, 2022) or indirectly by causing severe OE infiltration of immune cells, reduction of OSN neurogenesis, or disruption on the OSN expression of odorant receptors (Torabi et al., 2020; Finlay et al., 2022; Zazhytska et al., 2022). All direct and indirect damage to SCs can lead to a deficit in olfaction (Cooper et al., 2020; Tsukahara et al., 2023).

As COVID-19 progresses, a variety of neurological symptoms develop in up to 45% of patients (Priyal et al., 2023), suggesting that SARS-CoV-2 perturbs neuronal function. However, although the neuroinvasive potential of SARS-CoV-2 was proposed at the outset of the pandemic (L. Mao et al., 2020; Li et al., 2020; Moriguchi et al., 2020; Varatharaj et al., 2020), solid evidence to demonstrate neuronal infection in the human tissue remains in dispute. Several reports present compelling evidence of neuronal infection (Paniz-Mondolfi et al., 2020; Ramani et al., 2020; Burks et al., 2021; de Melo et al., 2021; Meinhardt et al., 2021; Song et al., 2021; Cappelletti et al., 2023; Yang et al., 2024), while others reported no direct infection of neurons (Brann et al., 2020; Pellegrini et al., 2020; Butowt et al., 2021, 2023; Khan et al., 2021; Finlay et al., 2022; Zazhytska et al., 2022; Luczo et al., 2024). Similarly, studies in laboratory animals report both the presence (Golden et al., 2020, 2022; Oladunni et al., 2020; Zheng et al., 2021; Beckman et al., 2022; Olivarria et al., 2022; Seehusen et al., 2022; Vidal et al., 2022; Yu et al., 2022; Villadiego et al., 2023; Shimizu et al., 2024) and absence (Bryche et al., 2020; Ye et al., 2021; Kishimoto-Urata et al., 2022; Verma et al., 2022) of neuronal infection. The possible role of brain infection of SARS-CoV-2 contributing to olfactory deficits in humans remains controversial (R. Choi et al., 2022). For example, Khan et al. (2021, 2022) found no evidence of infection in the CNS in a postmortem analysis of 68 individuals who died acutely diagnosed with SARS-CoV-2. However, the infection of the olfactory SC (cf. Khan et al., 2021) is widely accepted as an entry route that subsequently compromises odor receptor expression in sensory neurons (Frere et al., 2022; Zazhytska et al., 2022). Reports have also provided evidence of CNS infection in animals, including the olfactory system (OS) in hamsters (Frere et al., 2022), though the precise mechanisms providing access of SARS-CoV-2 into the brain remain under study. These include a hematopoietic route via infected macrophages or lymphocytes that extravasate the blood–brain barrier (BBB) or through breakdowns in the vasculature (Awogbindin et al., 2021; Kumari et al., 2021).

A definitive pathology that explains the diverse neurological symptoms, particularly in the OS, is still under debate. Some scientists argue that direct damage by SARS-CoV-2 to peripheral or central neurons, compromised myelin, and affected adult neurogenesis are still potential explanations for some of the symptoms of COVID-19 (Esposito et al., 2022, 2023; Fernandez-Castaneda et al., 2022; Lyoo et al., 2022; Stein et al., 2022; Ziuzia-Januszewska and Januszewski, 2022), although Monje and Iwasaki (2022) note that neuroinvasive infection in humans is rare.

To shed some light into the mechanisms of SARS-CoV-2 infections in olfactory pathways, we analyzed the spread of infection and neuroinflammation in the OS in K18hACE2 mice which can partially recapitulate the disease as seen in severely affected COVID-19 patients. Mice were infected with a previously determined dosage of virus that guaranteed the presence of pathology in brain tissues (Yinda et al., 2021; Dong et al., 2022; S. Choi et al., 2024). Using this experimental design, we addressed (1) the infection of the virus throughout the peripheral and central OS; (2) the characterization of infected cells; (3) the neuroinflammation caused by activated microglia; and (4) the effect on myelin along the olfactory myelinated tracts: the lateral olfactory tract (LOT) and the anterior commissure (AC).

## Materials and Methods

### Animals

All experiments were performed using 8-week-old male B6.Cg-Tg(K18-ACE2)2Prlmn/J mice (common name, K18hACE2; McCray et al., 2007) obtained from Jackson Laboratory (jax.org/strain/034860). These mice express human angiotensin I-converting enzyme 2 (hACE2) under the regulation of the epithelial cell keratin-18 (K18) promoter. Animals were raised and maintained in a BSL3 facility at Yale University. All protocols were approved by Yale University Animal Care and Use Committee.

### Generation of SARS-CoV-2 stocks

SARS-CoV-2 isolate hCOV-19/USA-WA1/2020 (NR-52281) was obtained from BEI Resources and subsequently amplified using VeroE6 cells overexpressing ACE2 and TMPRSS2 as previously described (T. Mao et al., 2022). Infectious viral titers were then determined by the plaque assay using Vero E6 cells overexpressing hACE2 and TMPRSS2.

### SARS-CoV-2 intranasal inoculation

Mice were anesthetized using 30% *v*/*v* isoflurane diluted in propylene glycol and subsequently intranasally inoculated with 5 × 10^3^ plaque-forming unit SARS-CoV-2 in 50 μl (25 µl per nostril) using a 200 μl pipette.

### Tissue processing and immunostaining

Seven days from infection, most animals lost 20% of body weight and became lethargic, meeting the euthanasia criteria. To obtain the OEs and brain tissues, animals were anaesthetized using a mixture of ketamine (50 mg/kg) and xylazine (5 mg/kg), injected intraperitoneally, and perfused first with 20 ml of PBS followed by 20 ml of ice-cooled 4% paraformaldehyde (PFA) in PBS. The heads were removed and fixed in 4% PFA overnight on a rotator. Then, entire brains were dissected from skulls and transferred to 30% sucrose in PBS for cryoprotection. OEs were first decalcified by immersing the entire nose containing the nasal cavity on 0.5 M EDTA for 1 month, then washed several times with PBS until the pH was neutralized, and finally transferred to a 30% sucrose in PBS for cryoprotection. Both OE and brains were embedded in Tissue-Tek optimal cutting temperature (OCT) compound (Thermo Fisher Scientific, catalog #4585) before being frozen for cryosectioning. Using a Reichert Frigocut cryostat (E-2800), we collected 25 µm serial sections on Fisherbrand ColorFrost Plus slides (Thermo Fisher Scientific) at the coronal plane, then dried using a slide warmer at 55°C, and stored at −80°C until use.

For immunohistochemistry (IHC), sections were thawed in a slide warmer at 60°C for 20 min and then washed with PBS to remove the OCT compound. Next, slides were immersed into a 0.01 M citrate buffer (pH 6.0) at 65°C for 35 min, followed by a 5 min incubation in ice-cooled 0.01 M citrate buffer, pH 6.0, to perform an antigen unmasking. Slides were placed horizontally in a humid chamber, and sections were washed three times, 10 min each with PBS + 0.1% Triton X-100 (Millipore Sigma, catalog #x100; PBST) before blocking for 1 h at room temperature (RT), with a solution made of PBST supplemented with 5% normal donkey serum (SouthernBiotech, catalog #0030-01) and 0.01% of protease-free bovine serum albumin (Sigma-Aldrich, catalog #A3059). Primary antibodies were diluted in 10% diluted blocking solution (Table 1), and sections were incubated with them overnight at 4°C. Slides were washed three times with PBST for 10 min, and appropriate secondary antibodies (Table 1), supplemented with 1 µg/ml of DAPI (Invitrogen, catalog #1306), were incubated for 2 h at RT. Finally, sections were washed three times for 10 min each with PBST, and slides were mounted with Mowiol 4-88 (Sigma-Aldrich, catalog #81381) prepared in 0.1 M Tris–glycerol.

**Table 1.** Primary and secondary antibodies

Antigen	Primary Ab	Source (catalog #)	Dilution	Secondary Ab	Source	Dilution
CNPase	Goat Poly.	Novus Biologicals (NBP3-05551) RRID: AB_3076521	1:1,000	Donkey anti-goat IgG Alexa Fluor 647	Thermo Fisher Scientific	1:1,000
Cytokeratin 8	Rabbit clone EP1628Y	Abcam (ab53280) RRID: AB_869901	1:300	Donkey anti-Rb IgG Alexa Fluor 488	Thermo Fisher Scientific	1:1,000
CLEC7A (Dectin-1)	Rat IgG2a, κ	InvivoGen (mabg-mdect) RRID: AB_2753143	1:30	Donkey anti-rat IgG Alexa Fluor 555	Thermo Fisher Scientific	1:1,000
Ctip2	Rat IgG2a	Abcam (ab18465) RRID: AB_2064130	1:500	Donkey anti-rat IgG Alexa Fluor 488 and 647	Thermo Fisher Scientific	1:1,000
IBA1	Rabbit Poly.	FUJIFILM Wako Chemicals (016-20001) RRID: AB_839506	1:200	Donkey anti-Rb IgG Alexa Fluor 488	Thermo Fisher Scientific	1:1,000
OMP	Goat Poly.	FUJIFILM Wako Chemicals (019-22291) RRID: AB_664696	1:500	Donkey anti-goat Alexa Fluor 647	Thermo Fisher Scientific	1:1,000
Reelin recombinant	Rabbit clone EPR26278-30	Abcam (ab312310) RRID: AB_3076463	1:100	Donkey anti-Ms IgG Alexa Fluor 488	Thermo Fisher Scientific	1:1,000
SARS-CoV-2 Nucleocapsid	Mouse IgG	Sino Biological (40143-MM05) RRID: AB_2827977	1:100	Donkey anti-Ms IgG Alexa Fluor 555	Thermo Fisher Scientific	1:1,000
Sox2	Rat IgG2a, κ	Thermo Fisher Scientific (14-9811-82) RRID: AB_11219471	1:500	Donkey anti-rat IgG Alexa Fluor 488	Thermo Fisher Scientific	1:1,000
Tbr1	Rabbit Poly.	Abcam (ab31940) RRID: AB_2200219	1:500	Donkey anti-Rb IgG Alexa Fluor 488	Thermo Fisher Scientific	1:1,000

### Imaging, quantifications, and statistics

To analyze the morphology of microglial cells in response to the SARS-CoV-2 infection, we performed a fractal analysis similar to the methods reported before (Soltys et al., 2001; Meller et al., 2023). Individual microglia cells (IBA1^+^) were randomly selected in Layers 1 and 2 of the piriform cortex (PC) and imaged with a ZEISS LSM 900 confocal microscope using a 63× objective. Three images of an IBA1^+^ cells were selected per animal for 18 total images for analysis. Images from the Alexa Fluor 488 channel were preprocessed using ImageJ Macro to binarize the image and isolate ionized calcium-binding adapter molecule 1 (IBA1) stained pixels from the background. Using the paintbrush tool, adjacent cell processes and background noise were removed. The binary image was converted to an outline using the ImageJ outline tool. The FracLac ImageJ plugin was used to perform fractal analysis on the outline. Num G was set to 4, and the metrics, convex hull, and bounding circle options were checked. The fractal dimension (D_B_), which quantifies cell complexity, was recorded. Differences between control noninfected and SARS-CoV-2–infected groups were assessed using a Welch's unpaired *t* test in the GraphPad Prism 10.1.2 software. *P* ≤ 0.05 was considered statistically significant (Table 2). Data is presented as mean ± SEM.

**Table 2.** Statistical analysis with significance

Analysis/figure	Test	Statistical value	Pairwise comparisons	*p* value
Number of SARS-CoV-2 cells (Fig. 2)	Two-way ANOVA + Tukey's multiple-comparison test	Mean diff. −851.7 95.00% CI of diff. −1,601 to −102.5	PC - LOT vs L1	0.0202
		Mean diff. −804.3 95.00% CI of diff. −1,554 to −55.13	PC - L1 vs L2	0.0309
		Mean diff. −964.2 95.00% CI of diff. −1,920 to −8.442	TuS - L1 vs L2	0.0476
Fractal analysis (cell morphology; Fig. 3)	Welch's unpaired *t* test	*t* = 3.746, df = 12.58	Control vs infected	0.0026
Macrophages counts in the OE (Fig. 4)	Multiple unpaired *t* tests	*t* = 2.917, df = 4.000	IBA1^+^ in septum	0.0434
Microglia counts in the OB (Fig. 5)	Multiple unpaired *t* tests	*t* = 5.104, df = 4.000	IBA1^+^ total (average)	0.006963
		*t* = 3.147, df = 4.000	IBA1^+^/Clec7A^+^ total (average)	0.034601
		*t* = 3.992, df = 4.000	IBA1^+^ in ONL	0.016240
		*t* = 3.955, df = 4.000	IBA1^+^ in GL	0.016746
		*t* = 5.812, df = 4.000	IBA1^+^/Clec7A^+^ in GL	0.004362
		*t* = 3.541, df = 4.000	IBA1^+^ in EPL	0.023985
		*t* = 3.989, df = 4.000	IBA1^+^ in GCL	0.016275
		*t* = 2.878, df = 4.000	IBA1^+^ in RMS	0.045123
Microglia counts in PC and TuS (Fig. 6)	Multiple unpaired *t* tests	*t* = 12.46, df = 4.000	IBA1^+^ in PC - total (average)	0.000239
		*t* = 8.485, df = 4.000	IBA1^+^ in PC - LOT	0.001058
		*t* = 10.85, df = 4.000	IBA1^+^ in PC - L1	0.000409
		*t* = 19.35, df = 4.000	IBA1^+^ in PC - L2	0.000042
		*t* = 9.483, df = 4.000	IBA1^+^ in PC - L3	0.000690
		*t* = 6.632, df = 4.000	IBA1^+^ in TuS - total (average)	0.002681
		*t* = 4.255, df = 4.000	IBA1^+^ in TuS - L2	0.013110
		*t* = 4.834, df = 4.000	IBA1^+^ in TuS - L3	0.008437
Average pixel intensity CNPase (Fig. 7)	Multiple unpaired *t* tests	*t* = 6.042, df = 4.000	CNPase pixels LOT	0.003785
		*t* = 5.229, df = 4.000	CNPase pixels AC	0.006389

To quantify the number of cells infected with SARS-CoV-2, we used the antibody that recognizes the nucleocapsid protein of the virus (SARS-CoV-2-NC^+^). To study inflammation, we used the staining for IBA1^+^ (microglial cells), as well as IBA^+^/Clec7A^+^, to determine the phagocytic-activated microglia. All images were acquired in a ZEISS LSM 800 confocal microscope using the 10×/20× objectives along the different OS areas: (1) the OE; (2) the olfactory bulb (OB); (3) the anterior PC; and (4) the tubular striatum (TuS), formerly the olfactory tubercle (Wesson, 2020). The OE was divided into two subsections, the septum and turbinates, and cells were quantified within the area from the edge of the epithelium to the lamina propria in randomly selected sections along the anterior to posterior axis. The OB was divided into its constitute layers, the olfactory nerve layer (ONL), glomerular layer (GL), external plexiform layer (EPL), mitral cell and internal plexiform layers (MCL-IPL) combined, granule cell layer (GCL), and the bulbar part of the rostral migratory stream (RMS), and quantified across the medial–lateral plane. The PC was divided into and quantified within its four layers: the LOT and Layers 1–3. The TuS was divided into and quantified within its three layers (1–3). The number of cells expressing the different markers were manually counted using ImageJ on 20× magnification confocal images. Cell counts were divided by their respective areas to calculate the average count per area. For each marker, images of the respective OS areas were captured across three consecutive sections separated by 250 µm. Images were taken in a total of three mice (*N* = 3) per group (noninfected controls vs SARS-CoV-2). Raw data were adjusted to represent the number of cells per mm^2^ before the statistical analysis. Statistical comparisons between the regions/layers for each olfactory area were made conducting a two-way ANOVA for SARS-CoV-2 quantifications and multiple unpaired *t* tests for IBA^+^ and IBA^+^/Clec7A^+^ using the GraphPad Prism 10.1.2 software. *P* ≤ 0.05 was considered statistically significant (Table 2). Data is presented as mean ± SEM.

The effect on myelin was studied in the main olfactory tracts: the AC and the LOT. Images were taken in the coronal plane with a ZEISS LSM 900 confocal microscope using a 20× objective. Two and three sections per animal were obtained for the AC and LOT, respectively. Myelin damage was measured by analyzing the 2′,3′-cyclic-nucleotide 3′-phosphodiesterase (CNPase) labeling intensity, which is an indirect indicator of myelin integrity. Images were processed using ImageJ Macro to binarize the image and isolate CNPase stained pixels from the background. Using a fixed area within the AC and LOT, the intensity of all CNPase pixels was measured and averaged to obtain the average intensity of the stained pixels. Statistical comparisons were made running a multiple unpaired *t* test using the GraphPad Prism 10.1.2 software. *P* ≤ 0.05 was considered statistically significant (Table 2). Data is presented as mean ± SEM.

## Results

### Distribution of SARS-CoV-2–infected cells across the OS

We examined cells targeted by SARS-CoV-2 in the OS in K18hACE2 mice, which expresses human ACE2 under the cytokeratin-18 promoter. At 7 d postinfection, we quantified their distribution across the different regions of the OS. OSNs have the initial exposure to SARS-CoV-2 following intranasal inoculation of the virus, followed by three regions in the CNS: the OB, the PC, and the TuS, formerly known as the olfactory tubercle (Wesson, 2020; Fig. 1*A*).

The OE is anatomically separated into the medial septum and the lateral turbinates, the latter of which has been suggested to be more sensitive to viruses (Urata et al., 2021). Here, we found that SARS-CoV-2 nucleocapsid-positive cells were sparsely distributed in both the septum and the turbinates. Uninfected mice showed no labeling with SARS-CoV-2-nucleocapsid (NC; data not shown). Infected cells had a polygonal morphology resembling SC and occurred individually or in small clusters of ∼10 cells (Fig. 1*B*,*B*’). The numbers of SARS-CoV-2^+^ cells did not differ between the septum and turbinates (Fig. 1*b*). In the OB, the majority of SARS-CoV-2^+^ cells were found in the MCL-IPL, where the largest projection neurons of the OB (mitral cells) are located (Fig. 1*C*,*c*). Fewer cells were found in the superficial ONL, as well as in the region of the GL and EPL where tufted cells, the second population of OB projection neurons, are located (Fig. 1*C*,*c*). In the EPL, some blood vessels were labeled for SARS-CoV-2 nucleocapsid (Fig. 1*C*, arrowheads), suggesting that endothelial cells were also targeted by the virus.

The cortical (PC) and subcortical (TuS) regions of the OS are the main areas of the brain receiving direct input from the OB. Both are arranged in layers and are innervated by the axons coming from the OB that form the LOT. In PC, these layers are as follows: a plexiform Layer 1, where the LOT axons make synapses with the apical dendrites of projection neurons; a densely packed Layer 2, where the projection neuron somata are located; and a thicker but less dense Layer 3 (Martin-Lopez et al., 2019a,b). Here, most infected cells were pyramidal/projection neurons located in Layers 2 and 3 (Fig. 1*D*,*d*), resembling the infections reported in neocortical pyramidal neurons (Golden et al., 2020; Vidal et al., 2022). Deep into the LOT, some SARS-CoV-2^+^ were occasionally observed in the superficial Layer 1 (Fig. 1*D*), likely belonging to local circuit horizontal cells (Neville and Haberly, 2004). In TuS, most SARS-CoV-2^+^ cells corresponded to projection neurons from Layers 2 and 3 (Fig. 1*E*,*e*).

Interestingly, the cytoarchitecture and morphology of all OS regions remained intact in infected animals, as previously reported in laboratory animals (Dedoni et al., 2022), suggesting that damage to the OS is likely concentrated at the molecular level. The mice employed in this study express the hACE2 protein under the control of the K18 promoter. Expression of K18 is found predominately in epithelial cells and not the brain (Abe and Oshima, 1990; McCray et al., 2007; Dedoni et al., 2022). Thus, the expression of hACE2 in the brain is almost undetectable. Nevertheless, our results established that SARS-CoV-2 did infect SC in the periphery and neurons across all areas of the central OS, suggesting the likely neurotropism of this virus under certain conditions of severe infection.

### Characterization of SARS-CoV-2–infected cells

**Figure 2.**
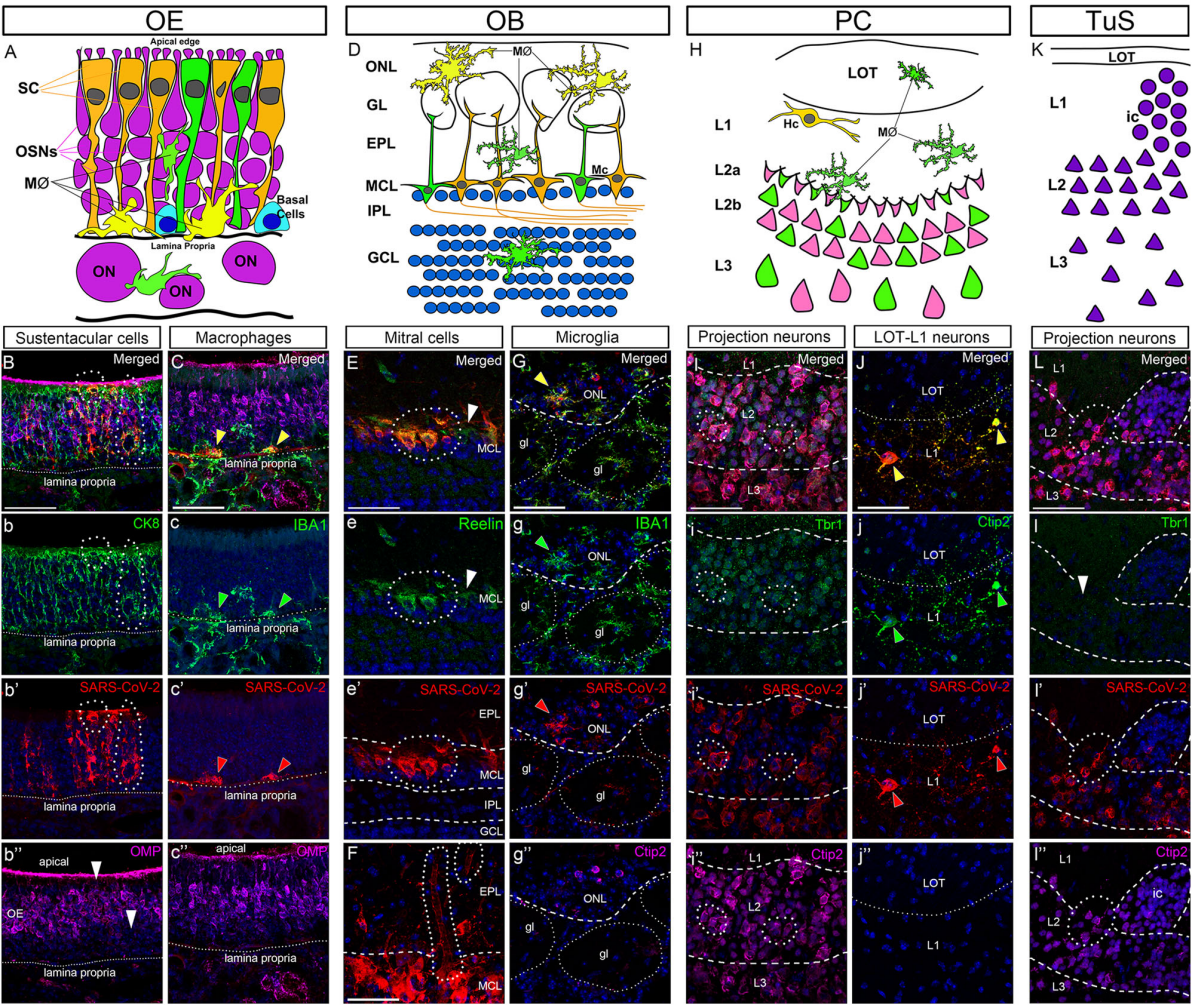
Molecular characterization of SARS-CoV-2–infected cells. ***A***, Diagram illustrating the main cell types of the OE. ***B***–***b*’’**, IHC in the OE detecting the SC marker CK8 (***b***, green), SARS-CoV-2-NC (***b*’**, red) and OSN marker OMP (***b*’’**, magenta). SARS-CoV-2-NC^+^ cells colocalize with CK8 (***B***) but not with OMP (***b*’’**, arrowheads). ***C***–***c*’’**, IHC in the OE staining for the macrophage marker IBA1 (***c***, green), SARS-CoV-2-NC (***c*’**, red), and OMP (***c*’’**, magenta), showing infected macrophages lying in the lamina propria (arrowheads). ***D***, Diagram illustrating the layers and main cell types of the OB. ***E***–***e*’**, IHC in the OB staining for Reln (***e***, green) and SARS-CoV-2-NC (***e*’**, red). All SARS-CoV-2-NC^+^ cells in the MCL express Reln (***E***, ***e***), confirming their phenotype as infected mitral cells. ***F***, SARS-CoV-2-NC^+^ labeling in blood vessels from the EPL. ***G***–***g*’’**, IHC staining for IBA1 (***g***, green), SARS-CoV-2-NC (***g*’**, red), and Ctip2 (***g*’’**, magenta), showing infected microglial cells in the ONL (***G***–***g’***, arrowheads). ***H***, Diagram illustrating the layers and main cell types of the PC. ***I***–***i*’’**, IHC in PC staining for Tbr1 (***i***, green), SARS-CoV-2-NC (***i*’**, red), and Ctip2 (***i*’’**, magenta). All neurons from Layers 2 and 3 are found to coexpress SARS-CoV-2-NC^+^ with Tbr1 (***I***, ***i***, dotted lines) and Ctip2 (***I***, ***i*’’**, dotted lines) confirming they are projection neurons. ***J***–***j*’’**, IHC staining to detect Ctip2 (***g***, green) and SARS-CoV-2-NC (***g*’**, red) showing that Hc from the superficial Layer 1 are infected with SARS-CoV-2 (arrowheads). ***K***, Diagram illustrating the layers and of the TuS. ***L–l*’’**, IHC in TuS staining for Tbr1 (***l***, green), SARS-CoV-2-NC (***l*’**, red), and Ctip2 (***l*’’**, magenta) showing the absence of Tbr1 expression in the entire TuS and coexpression of SARS-CoV-2-NC with Ctip2 highlighting the virus targeting projection neurons in the TuS. Nuclei counterstained with DAPI (blue). CK8, cytokeratine-8; Ctip2, COUP-TF–interacting protein 2; gl, glomeruli; Hc, horizontal cell; ic, island of Calleja; IHC, immunohistochemistry; LOT, lateral olfactory tract; MØ, microglia; Mc, mitral cells; OB, olfactory bulb; OE, olfactory epithelium; OMP, olfactory marker protein; OSN, olfactory sensory neurons; PC, piriform cortex; RMS, rostral migratory stream; SC, supporting/sustentacular cells; Tbr1, T-Box brain transcription Factor 1; TuS, tubular striatum. Scale bar, 50 µm.

Next, we sought to investigate the cellular phenotypes of those cells that showed an affinity for SARS-CoV-2 infection throughout the different OS regions. The OE lines the posterior part of the nasal cavity and includes three primary epithelial cells (Harkema et al., 2006; Fig. 2*A*): (1) the OSNs; (2) SC; and (3) basal cells which are the OE neural stem cells. In mice, OSNs are characterized by their exclusive expression of olfactory marker protein (OMP) (Farbman and Margolis, 1980) and only 1 of the ∼1,200 odorant receptors (Vassar et al., 1993). The SCs are columnar epithelial cells that surround the OSNs and provide them with support and nutrients. SCs can be identified with the expression of cytokeratin-8 (CK8; Maurya et al., 2015). The basal cells lie next to the lamina propria and are subdivided into horizontal basal cells that express cytokeratin-5 (Maurya et al., 2015) and globose basal cells that express Sox2 (Guo et al., 2010). Our results showed that SARS-CoV-2^+^ cells in the OE belonged to two different populations: (1) cells that exhibited an elongated and polygonal cell shape that extended from the apical surface to the lamina propria of the OE, expressing CK8 but not OMP (Fig. 2*B*–*b*’’), identified as SC, and (2) macrophages lying over the lamina propria that expressed IBA1, a marker characteristic of the monocyte/macrophage lineages (Fig. 2*C*–*c*’’). Basal cells expressing Sox2 were not infected with SARS-CoV-2 (data not shown). The preferential infection of SC by SARS-CoV-2 aligns with previous reports in both laboratory animals and humans (Cantuti-Castelvetri et al., 2020; Khan et al., 2021; Ye et al., 2021; Finlay et al., 2022; Verma et al., 2022; Zazhytska et al., 2022). However, we pioneered in finding infection only from macrophages in the OE lamina propria.

Across the OB layers (Fig. 2*D*), most SARS-CoV-2^+^ cells were found in the MCL-IPL which colocalized with the marker Reelin (Reln; Fig. 2*E*–*e*’). Reln is characteristic of OB projection neurons, mitral and tufted cells (M/Tc), which are found in the MCL-IPL and EPL (Martin-Lopez et al., 2011). Similar results were observed with those cells located in the deep GL and superficial EPL (data not shown). Reln has, however, been implicated in hyperinflammatory responses in epithelial cells in COVID-19 patients (Calvier et al., 2023). Since there is evidence for ACE2 coexpression with furin in the GL and MCL (Ueha et al., 2021), our data align with the hypothesis that under the specific conditions of severe disease, SARS-CoV-2 is able to infect M/Tc, possibly using ACE2/furin as entry protein receptors. In the ON, where OSN axons enter the OB, some SARS-CoV-2^+^ cells were found coexpressing the marker IBA1 and were identified as infected microglia (Fig. 2*G–g*’’). Blood vessels were frequently labeled with SARS-CoV-2-NC in the EPL (Fig. 2*F*).

In PC and TuS, SARS-CoV-2 was found labeling only neurons and not microglial cells. The PC is a trilaminar cortex deep into the LOT and acts as the primary and associational olfactory cortex (Fig. 2*H*). Here, projection neurons are glutamatergic and express Tbr1 and Ctip2 (Martin-Lopez et al., 2019a,b). Both markers were coexpressed with SARS-CoV-2-NC^+^ in neurons of Layers 2 and 3 in K18hACE2 mice (Fig. 2*I*–*i*’’). In Layer 1, some horizontal cells were also infected with SARS-CoV-2, and although they are presumed to be GABAergic interneurons (Shepherd et al., 2021), they also expressed the marker Ctip2 (Fig. 2*J*–*j*’’). The expression of Ctip2 by GABAergic neurons is characteristic of projection neurons in the TuS Layers 2 and 3, which do not express the cortical marker Tbr1 (Martin-Lopez et al., 2019b). Our data in the TuS demonstrated that SARS-CoV-2^+^ cells were located in Layers 2 and 3 and in the islands of Calleja, all expressing Ctip2 (Fig. 2*K*,*L*–*l*’’).

Collectively, these data confirmed that under conditions of acute disease in the K18hACE2 mice, SARS-CoV-2 targets SC and macrophages in the OE, while it showed neurotropism for projection neurons in the OB, PC, and TuS. Some microglial cells were also labeled with SARS-CoV-2-NC in the ONL and blood vessels in the EPL inside the OB. Since OSNs were not labeled with SARS-CoV-2-NC in the OE, which are the only cells connected to M/Tc through the olfactory nerve, axonal transport seems unlikely, and we support the hypothesis of a different entry route into the CNS likely using a hematopoietic pathway (Awogbindin et al., 2021; Tsukahara et al., 2023).

### Microglial activation in response to SARS-CoV-2

During inflammation of the CNS, activated microglia proliferate in a process known as microgliosis (Zabel and Kirsch, 2013; Borst et al., 2021). After SARS-CoV-2 infections, microgliosis is well documented in both humans and laboratory animals (Poloni et al., 2021; Fernandez-Castaneda et al., 2022; Kaufer et al., 2022; Kishimoto-Urata et al., 2022; Olivarria et al., 2022; Dey and Bishayi, 2023). We first assessed the morphological changes associated with microgliosis by using a fractal analysis of individual cells in PC as previously described (Soltys et al., 2001; Meller et al., 2023). We searched for resting states, identified by small round cell bodies that extend highly branched thin processes (ramified microglia), and activated microglia, whose morphology become amoeboid and hypertrophic, characterized by large cell bodies with shorter thicker processes (Blackbeard et al., 2007; Lier et al., 2021). Microglia were identified with the marker IBA1, characteristic of monocyte–macrophage lineages.

**Figure 3.**
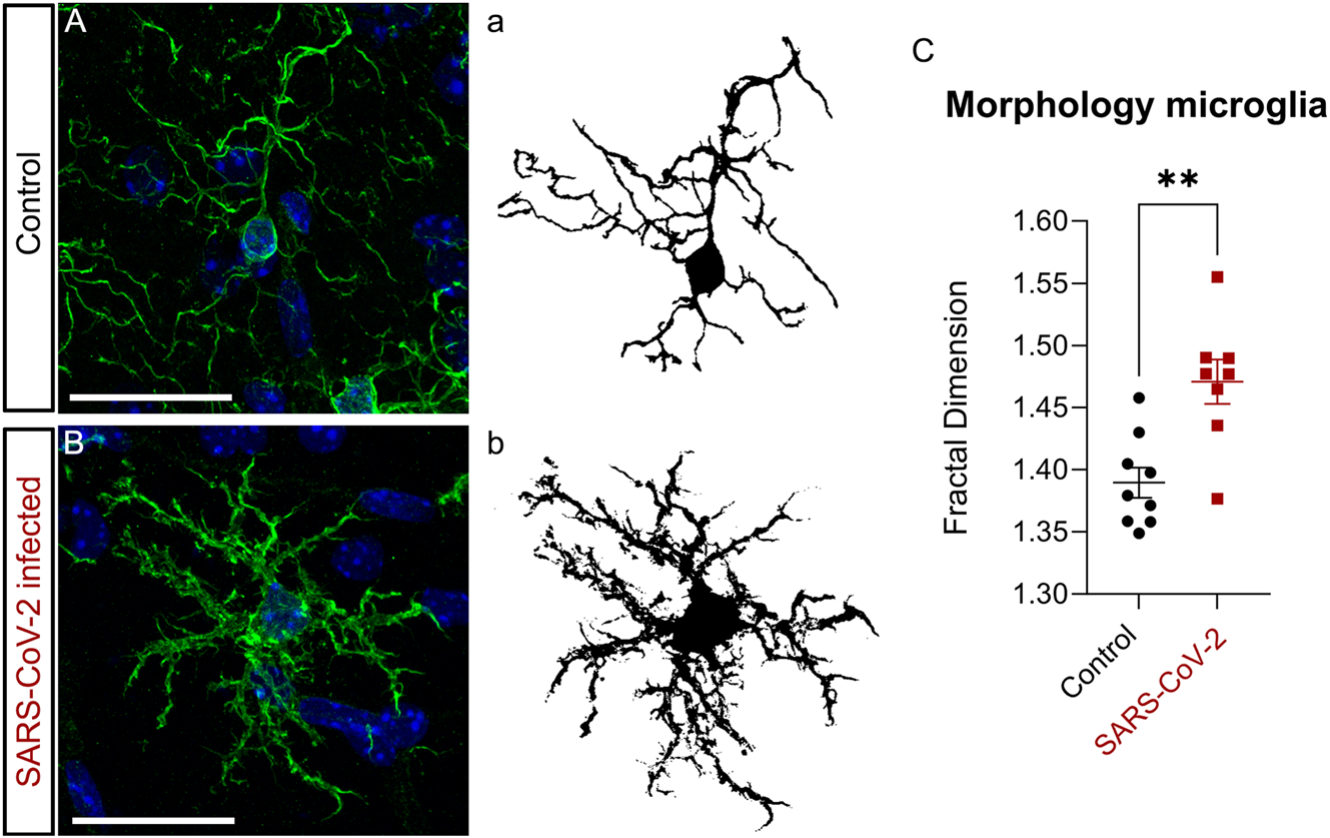
Morphology of activated microglia. Immunostaining for the microglial marker IBA1 (green) with nuclei counterstained with DAPI (blue). ***A***, ***a***, Morphology of microglia in control animals. ***B***, ***b***, Morphology of microglia in infected animals. ***C***, Microglia changes in morphology in response to SARS-CoV-2 infection to show a statistically significant increase in the fractal dimension in infected mice. Statistics: unpaired *t* test with ** = *p* < 0.01 (Table 2). Scale bar, 25 µm.

After 7 d of infection with SARS-CoV-2, most microglial cells in PC showed an activated state (Fig. 3*A*–*C*) as described in the neocortex (Villadiego et al., 2023). This effect may be unique for coronaviruses, since brain infections with other viruses, such as influenza, do not induce changes in microglia morphology (Dusedau et al., 2021). In the OE, changes in macrophage morphology were not analyzed since those changes have not yet been fully characterized in vivo, and it is believed that they change gradually in a spectrum of different elongated states (McWhorter et al., 2013).

### Microgliosis and macrophage activation in the OS induced by SARS-CoV-2

Next, we studied macrophages (in the periphery) and microglia (within the CNS) proliferation by quantifying their numbers along the different OS regions, as well as their phagocytic activity. Phagocytic macrophages/microglia were assessed by labeling with the marker C-type lectin domain family 7 member A or Dectin-1 (Clec7A; Krasemann et al., 2017). Infectious diseases targeting neural regions lead to activation of the monocyte/macrophage lineages resulting in strong inflammation (Hoogland et al., 2015; Merad et al., 2022). Macrophages/microglial cells were identified with IBA1, while those actively phagocyting were identified with the coexpression of IBA1^+^/Clec7A^+^. We colabeled with SARS-CoV-2-NC to determine if inflammatory cells clustered around infected cells.

**Figure 4.**
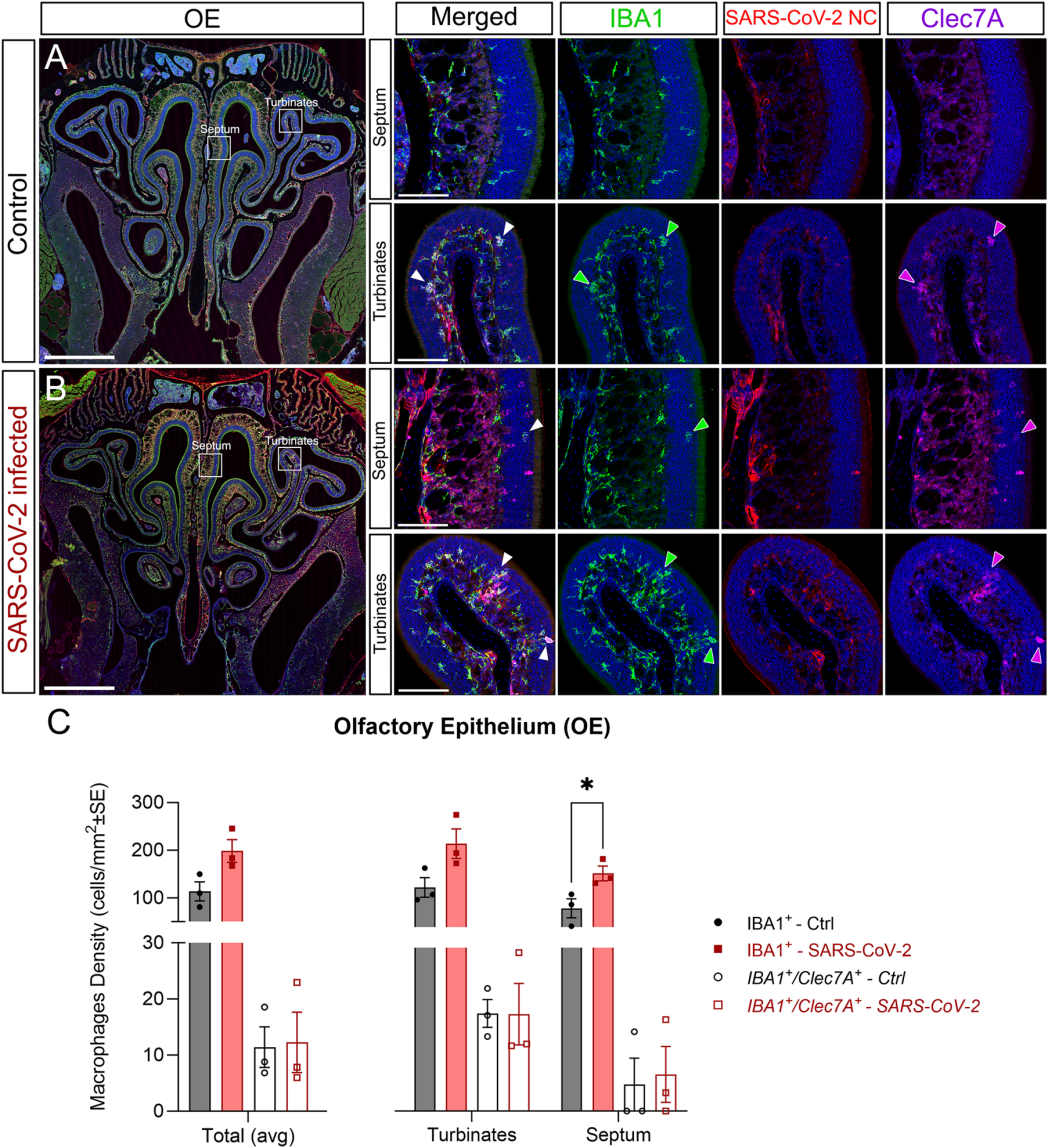
Microgliosis in the OE. Staining of the OE with the macrophage marker IBA1 (green), SARS-CoV-2 nucleocapsid (red), and active phagocytic macrophages Dectin-1 (Clec7A; magenta). Nuclei counterstained with DAPI (blue). ***A*** with insets, Representative images of the OE from control mice. ***B*** with insets, Representative images of the OE from SARS-CoV-2–infected mice. ***C***, Quantification of total macrophage cells (IBA1^+^) and phagocytic macrophages (IBA1^+^/Clec7A^+^) in the medial wall (septum) and turbinates. The number of combined IBA1+ cells between the septum and turbinates shows an increase that was statistically significant only in the septum (***C***), suggesting a higher vulnerability of this region to the infection. Statistics: multiple unpaired *t* test with * = *p* < 0.05 (Table 2). Scale bars: ***A*** and ***B***, 1 mm; septum and turbinate insets, 100 µm.

In the OE, there was an overall increase in the total number of macrophages that was significantly higher in the septum of infected mice (Fig. 4*C*). The increase in the turbinates was evident but not statistically significant (Fig. 4*C*). The number of phagocytic macrophages (IBA1^+^/Clec7A^+^) showed no statistically significant differences between controls and infected mice (Fig. 4*A*,*B*, arrowheads; *C*). The significant increase in the number of IBA1^+^ macrophages in the septum suggested this region may be more vulnerable to inflammation than the turbinates, even though both regions were equally infected by the virus (Fig. 1*b*). These data contrast with that of others who suggested a higher sensitiveness of the turbinates (Kaufer et al., 2022; Ueha et al., 2022). No clustering of macrophages was evident around SARS-CoV-2^+^ cells.

**Figure 5.**
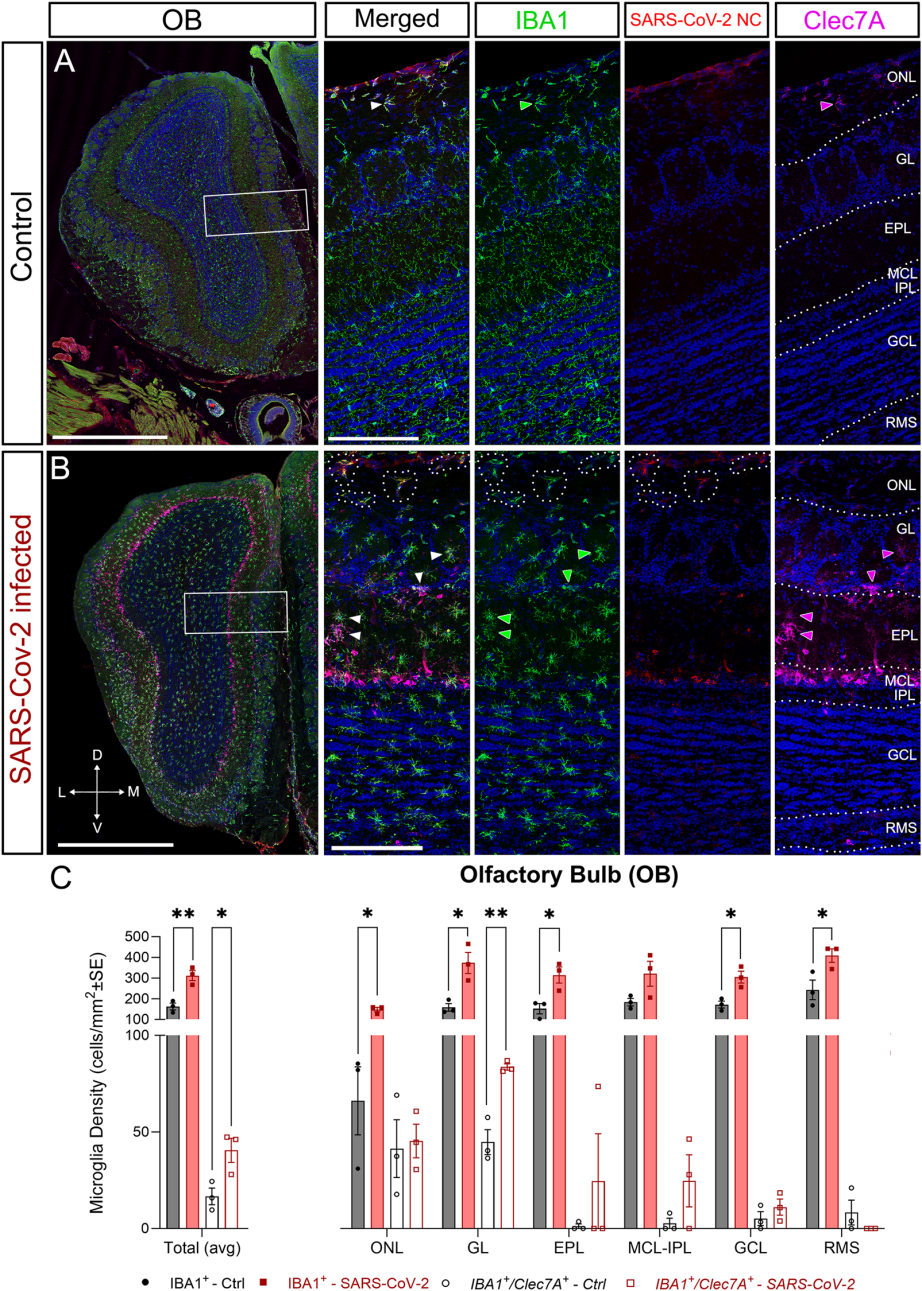
Microgliosis in the OB. Staining of microglia with IBA1 (green), SARS-CoV-2 nucleocapsid protein (red), and active phagocytic microglia with Dectin-1 (Clec7A; magenta). Nuclei counterstained with DAPI (blue). ***A***, Representative images of the OE from control mice. ***B***, Representative images of the OE from SARS-CoV-2–infected mice. Microglial cell bodies show a clear hypertrophy related to an activated response. Infected microglia are observed in the ONL (dotted lines). ***C***, Quantification of the microglia (IBA1^+^) and phagocytic microglia (IBA1^+^/Clec7A^+^) in the OB. Total numbers of microglia (IBA^+^) are statistically significant higher numbers in infected animals, suggesting a strong inflammation. This effect is replicated on each individual layer excepting in the MCL-IPL, which is the layer less affected by the microgliosis. Numbers of phagocytic microglia (IBA1^+^/Clec7A^+^) are significantly higher only in the GL, indicating that this layer is the most affected by inflammation. EPL, external plexiform layer; GCL, granule cell layer; GL, glomerular layer; IPL, internal plexiform layer; MCL, mitral cell layer; OB, olfactory bulb; ONL, olfactory nerve layer; RMS, rostral migratory stream (bulbar part). Statistics: multiple unpaired *t* test with * = *p* < 0.05; ** = *p* < 0.01 (Table 2). Scale bars: ***A*** and ***B***, 1 mm; high magnification insets, 200 µm.

In the OB, the total number of microglia (IBA1^+^) and phagocytic microglia (IBA1^+^/Clec7A^+^) was significantly higher in infected animals, suggesting an enhanced inflammatory response to the presence of the virus (Fig. 5*A*–*C*). Quantifications were made by OB layers to detect whether the inflammation in the OB occurred predominantly in those layers where we detected the virus (Fig. 1*C*,*c*). Unexpectedly, our data showed microglial numbers were significantly higher across all layers of the OB with the exception of the MCL-IPL (Fig. 5*C*). The number of phagocytic microglia was significantly higher in infected animals only in the GL (Fig. 5*C*). Infected microglia were also evident in the adjacent ONL (Fig. 5*B*, dotted lines), where OSN axons reach the OB. These data suggested that microgliosis was independent of the location of the infected cells.

**Figure 6.**
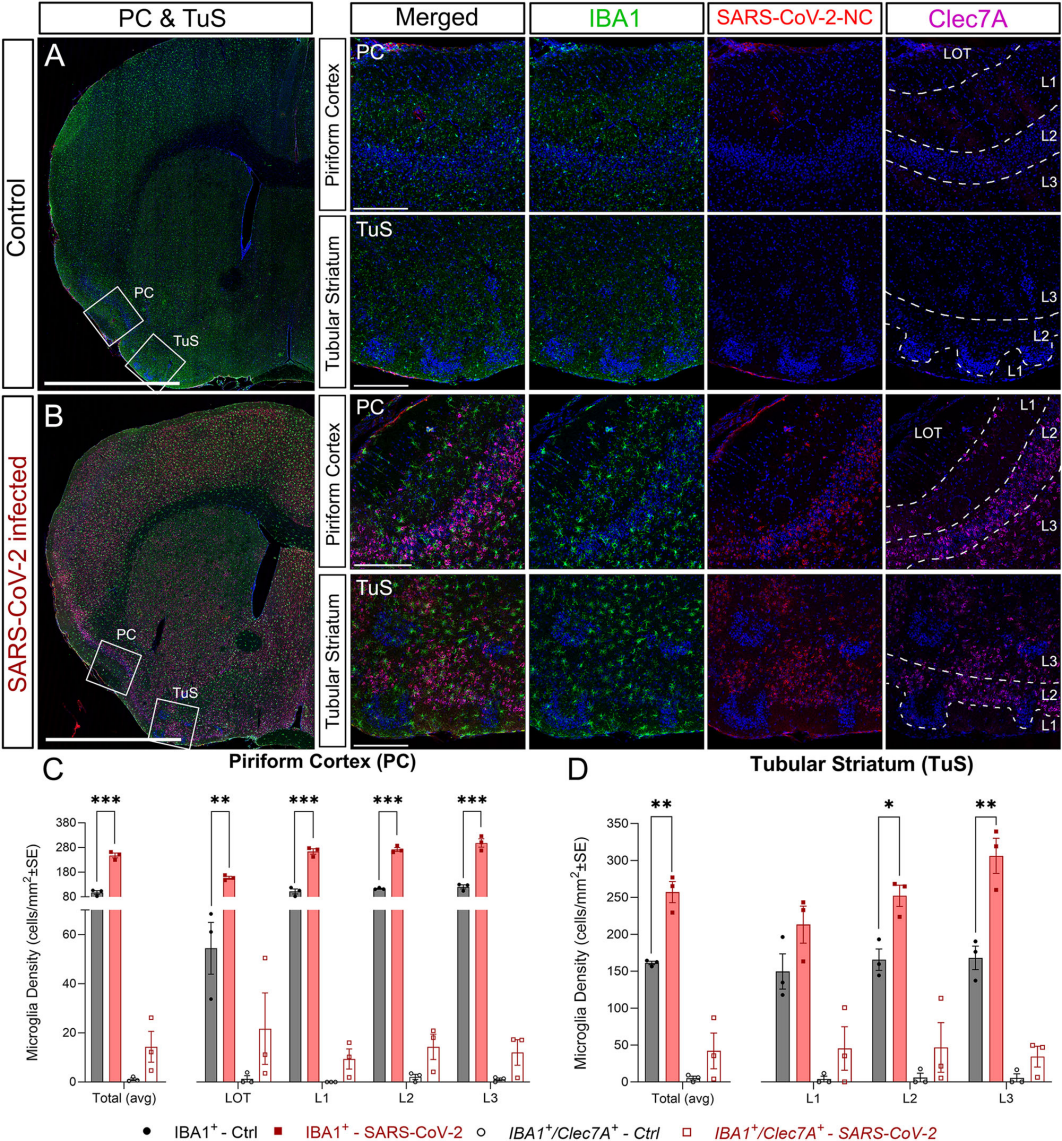
Microgliosis in piriform cortex and TuS. Staining of microglia with IBA1 (green), SARS-CoV-2 nucleocapsid (red), and active phagocytic macrophages with Dectin-1 (Clec7A; magenta). Nuclei counterstained with DAPI (blue). ***A***, Representative images of the PC and TuS from control mice. ***B***, Representative images of the PC and TuS from SARS-CoV-2–infected mice, where clear hypertrophy is observed in all cell bodies extending not only into the PC and TuS but also across the entire brain. ***C***, Quantification of microglia (IBA1^+^) and phagocytic microglia (IBA1^+^/Clec7A^+^) in PC. The numbers of microglia are statistically significantly higher in all layers of PC from infected animals proving the presence of strong inflammation. ***D***, Quantification of microglia (IBA1^+^) and phagocytic microglia (IBA1^+^/Clec7A^+^) in TuS. The numbers of microglia (IBA1^+^) were significantly higher in Layers 2 and 3 highlighting that inflammation affected the projection neuron layers. Numbers of phagocytic microglia (IBA1^+^/Clec7A^+^) are similar in both controls and infected mice. LOT, lateral olfactory tract; PC, piriform cortex; TuS, tubular striatum. Statistics: multiple unpaired *t* test with * = *p* < 0.05; ** = *p* < 0.01; *** = *p* < 0.001 (Table 2). Scale bars: ***A*** and ***B***, 1 mm; high magnification insets, 200 µm.

Finally, in PC and TuS, microgliosis was widely distributed. In PC we found a significant increase in the total number of microglial cells across all layers of PC, showing an evident change to an activated cell morphology (Fig. 6*A*_(PC)_, *B*_(PC)_, *C*). Unexpectedly, the number of phagocytic microglia was almost undetectable, showing a slight, but not significant, increase across the layers. Similarly, in TuS we observed microgliosis predominantly in the densely packed cell Layers 2 and 3 (Fig. 6*A*_(TuS)_, *B*_(TuS)_, *D*). Although we did not quantify cells in other areas of the brain, it was evident that microgliosis was widespread within the CNS of infected animals (Fig. 6*B*) as previously reported (Golden et al., 2022; Olivarria et al., 2022; Seehusen et al., 2022).

### Disruptions in myelin integrity caused by SARS-CoV-2

**Figure 7.**
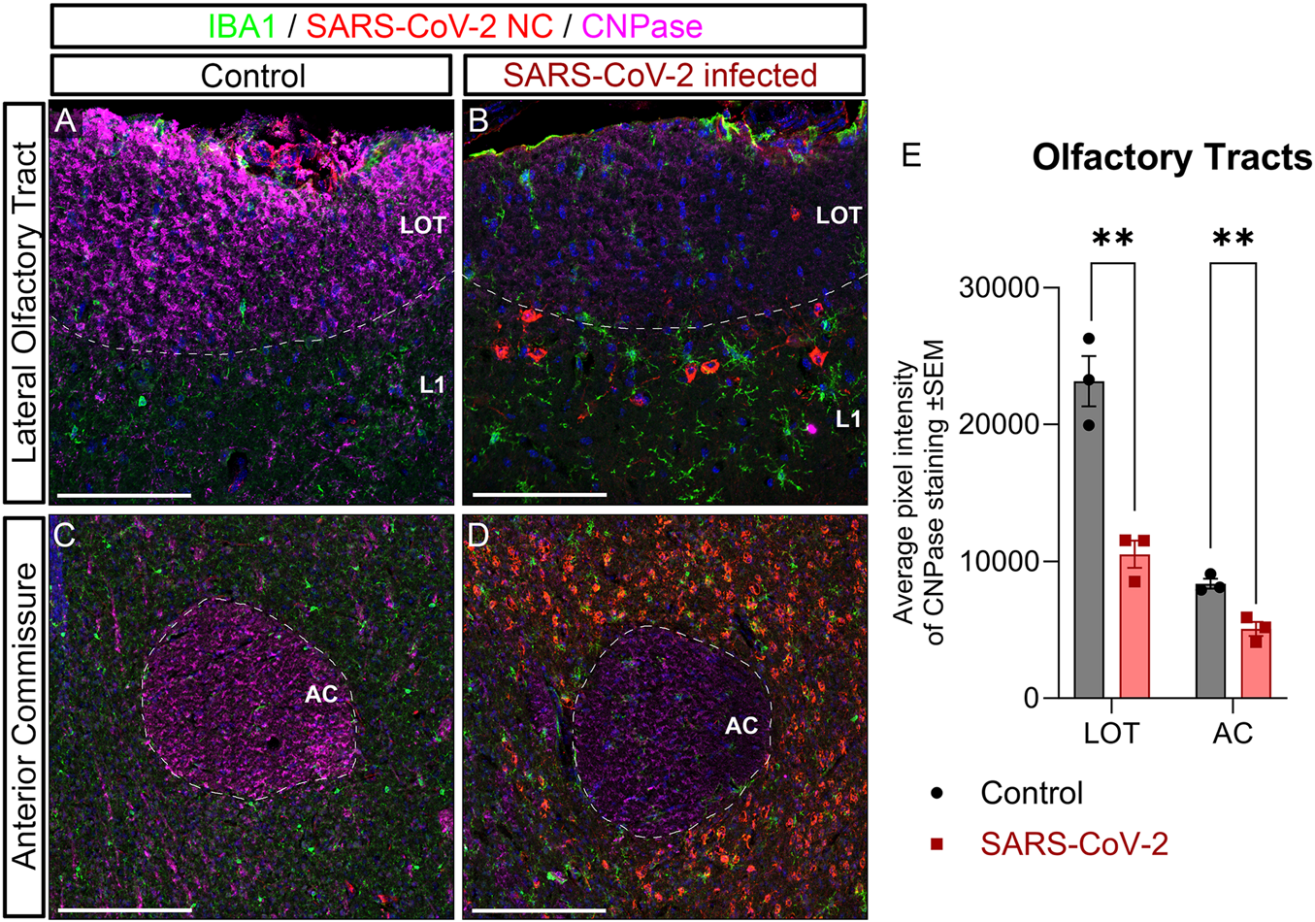
Myelination defects in the LOT and AC. ***A–D***, Staining of microglia with IBA1 (green), SARS-CoV-2 nucleocapsid (red), and CNPase (magenta). Nuclei counterstained with DAPI (blue). ***A***, Representative images of IHC staining in the LOT of control and infected mice. ***B***, Representative images of IHC staining in the AC of control and infected mice. ***E***, Quantification of the CNPase pixel intensity shows a statistically significant decrease in the intensity for both, the LOT and AC in SARS-CoV-2–infected mice compared with those in controls. AC, anterior commissure; LOT, lateral olfactory tract. Statistics: multiple unpaired *t* test with ** = *p* < 0.01 (Table 2). Scale bar, 100 µm.

In the CNS, the regulation of axon–oligodendroglia interactions is essential to maintain the integrity of the myelin sheaths. In mature myelinated tracts, the enzyme CNPase plays a critical role in maintaining a healthy myelin–axon interface (Lappe-Siefke et al., 2003). Some evidence suggests that SARS-CoV-2 has an impact on the brain myelin that could explain some of the neurological symptoms displayed by COVID-19 patients (Domingues et al., 2020; Poloni et al., 2021; Fernandez-Castaneda et al., 2022). In this work, we measured the CNPase levels in the two main OS myelinated tracts, the LOT and the AC, to examine myelin damages in the K18hACE2 mouse model of COVID-19. Our analyses revealed that there was a statistically significant reduction of CNPase in both the LOT and the AC (Fig. 7*A*–*E*) after 7 d of infection, suggesting a loss of myelin and altered axonal conduction.

## Discussion

The awareness of sense of smell during our daily life changed dramatically in 2019 with the emergence of a new strain of coronavirus that caused the COVID-19 disease. At the beginning of the pandemic, the number of people infected with SARS-CoV-2 that reported some level of olfactory dysfunction exceeded 80% (Cooper et al., 2020; Lechien et al., 2020). These numbers pointed to the OS as one of the main targets of the virus. Most evidence suggests that compromise of the SC is the main reason for direct or indirect damages to the OSNs that produce olfactory dysfunction (Khan et al., 2021, 2022; Finlay et al., 2022; Zazhytska et al., 2022; Ziuzia-Januszewska and Januszewski, 2022; Tsukahara et al., 2023). However, although the findings remain controversial, there are some data suggesting that SARS-CoV-2 can infect neurons in the OS and therefore contribute to the perturbation of olfactory function. To shed new light on this paradigm, we used the K18hACE2 mice to study the viral infection and inflammation in the OS, especially in the central regions OB, PC, and TuS (Yinda et al., 2021; Zheng et al., 2021; Dong et al., 2022; S. Choi et al., 2024).

Our analysis in the OE confirmed that SC are the only cell type susceptible to infection in the OE of K18hACE2 mice, consistent with reports of human pathology (Bryche et al., 2020; Cantuti-Castelvetri et al., 2020; Khan et al., 2021; Ye et al., 2021; Finlay et al., 2022; Verma et al., 2022; Zazhytska et al., 2022). These findings were expected since SCs are the only cells expressing the tandem proteins ACE2/TMPRSS2 used by SARS-CoV-2 as entry cell molecules (Bilinska et al., 2020; Brann et al., 2020; Fodoulian et al., 2020). Our data support the hypothesis that direct damage to OSNs is unlikely to explain loss of smell, as some have proposed (Sia et al., 2020; Meinhardt et al., 2021; Ziuzia-Januszewska and Januszewski, 2022; Shimizu et al., 2024). In addition to SARS-CoV-2 infection in the OE, we found that inflammation was more prominent in the septum compared with that in the turbinates unlike prior reports (Golden et al., 2020, 2022; Kumari et al., 2021; Ueha et al., 2022). We speculate that SC in the septum may exhibit different topographically defined molecular phenotypes as occurs with the OSNs (Zapiec and Mombaerts, 2020) that could make them to be more susceptible of infection. We also found that macrophages in the lamina propria of the OE were infected by SARS-CoV-2, but not those that were distributed throughout the thickness of the OE or internal to the lamina propria (Fig. 2*C*–*c*’). The ability of SARS-CoV-2 to infect cells from the monocyte/macrophage lineages is known (Jeong et al., 2022; Eberhardt et al., 2023), but here we demonstrate for the first time that only those macrophages aligned to the lamina propria were infected. IBA1^+^ cells arranged along the lamina propria were reported to be proximal to infected SC, but they were never infected with SARS-CoV-2 (Bourgon et al., 2022). In our tissues, none of these macrophages were found proximal to SC-infected cells that could explain a cell-to-cell transference of viral particles. This possibility would benefit from further research addressing the vulnerability of IBA^+^ cells in the OE to SARS-CoV-2 infections.

However, despite the damage to the epithelium, the question regarding the possibility of the virus infecting neurons within the CNS that could contribute to olfactory deficits, particularly those causing long-term loss of olfaction, remains unanswered. While Meinhardt et al. (2024) speculate on indirect effects on neurons (Meinhardt et al., 2024), there is also evidence that support direct neuronal damage caused by the virus. For example, Esposito et al. (2022, 2023) reported anomalies in cortical connectivity and function following SARS-CoV-2 infections. In support of a CNS pathology, SARS-CoV-2 RNA has been reported in brain biopsies from COVID-19 patients (Matschke et al., 2020; Meinhardt et al., 2021). Finally, damage in the myelin sheath of CNS axons is well documented both here and previously and could contribute to neurological symptoms including impaired olfactory function (Garg et al., 2021; Fernandez-Castaneda et al., 2022). These data represent examples of damages in the CNS that, at least in some patients, involve some degree of neuronal infection by SARS-CoV-2 that may host replicating viral particles inside the cells.

Interestingly, within the OB, the virus infected the projection neurons M/Tc, which was most likely the result of their coexpression of furin with murine ACE2, which have been suggested to be alternative SARS-CoV-2 entry receptors (Ueha et al., 2021). It seems reasonable to speculate and open for discussion the possibility that the infection of other projection neurons throughout the brain was associated with the expression of other proteins used by the virus as entry receptors that are independent of ACE2 or TMPRSS2 (Iadecola et al., 2020; Zhao and Gao, 2024).

In accordance with previous work, we found significantly higher numbers of microglia in all OS regions (OB, PC, and TuS) suggesting widespread neuroinflammation (Awogbindin et al., 2021; Savelieff et al., 2022; Vidal et al., 2022). However, others have reported an increase in the OB but not in PC (Kaufer et al., 2022); an increase only in PC (Carossino et al., 2022); no changes at all (Fernandez-Castaneda et al., 2022); or reductions in the numbers of microglia (Jeong et al., 2022). While the question remains to be resolved, it seems that the most parsimonious explanation would include microgliosis accompanying the SARS-CoV-2 infections and accounting, at least in part, for the associated neurological symptoms. Limitations of this study include the use of transgenic mice as a model for human disease, which may not recapitulate precisely the pathology seen in infected humans, and the use of only the ancestral isolate of SARS-CoV-2, which may not represent the pathology of more recent variants of concern.

In summary, we found that SARS-CoV-2 infections in the K18hACE2 mouse infected SC and lamina propria macrophages in the OE, but not OSNs. In the CNS, SARS-CoV-2 infected microglial cells in the ONL of the OB and projection neurons in all OS regions (OB, PC, and TuS). The most obvious alteration that we observed was a widespread microgliosis throughout the brain and damage to integrity of the myelin in the LOT and the AC. However, there were no evident disturbances to the cytoarchitecture in these regions suggesting that damages may reside at the molecular level. Our data support the hypothesis that the neuronal infection likely occurred through infected microglial cells used as a “Trojan horse” to infect neurons as has been previously proposed (Awogbindin et al., 2021). Collectively, these findings contribute to our understanding of the olfactory deficits in both humans and animal models with SARS-CoV-2 infections. Therefore, therapies designed to target the virus entry points, such as the use of vaccines that induce a humoral response producing blocking antibodies of the spike protein, seems to be the safest approach to ensure protection against brain infection.

## Synthesis

Reviewing Editor: Matthew Grubb, King's College London Decisions are customarily a result of the Reviewing Editor and the peer reviewers coming together and discussing their recommendations until a consensus is reached. When revisions are invited, a fact-based synthesis statement explaining their decision and outlining what is needed to prepare a revision will be listed below. The following reviewer(s) agreed to reveal their identity: Stuart Firestein. Note: If this manuscript was transferred from JNeurosci and a decision was made to accept the manuscript without peer review, a brief statement to this effect will instead be what is listed below.

Synthesis:

Both Reviewers agree that the data are solid. After consultation, we concurred that descriptions of the data, contextualisation of findings, and discussion of potential implications need to be altered throughout the manuscript. Specifically, great care needs to be taken to include and clarify previous relevant findings using different animal models and human tissue approaches. In addition, the manuscript needs to be absolutely explicit about the K18hACE2 mouse model, including the ways in which ACE2 expression patterns and COV2 infection characteristics differ from those in human. Any claims for clinical relevance need to be extremely carefully framed, with all caveats clearly stated. Please also respond to all of the Reviewers' individual concerns listed below.

Reviewer 1:

In this manuscript, the authors used a line of transgenic mice expressing human ACE2 (the receptor for the deadly virus SARS-CoV-2) to study the infection of SARS-CoV-2 in the olfactory system, in both the peripheral and the central projections. Loss of olfaction was one of the prominent symptoms of SARS-CoV-2 infection at the beginning of the pandemic. Intriguingly, the olfactory sensory neurons directly responsible for detecting the olfactory stimulus are not infected by the virus. Consistent with many previous reports, the authors also showed that SARS-CoV-2 targeted the sustentacular cells in the olfactory epithelium, validating their experimental approach. They further showed convincing and solid evidence that numerous projection neurons are infected in multiple higher olfactory centers. More importantly, they observed microgliosis (a reaction of microglia to pathogens) in several regions of the central olfactory systems, suggesting neuroinflammation may account for the neurological symptoms after SARS-CoV-2 infection.

There are a few points the authors could address to improve the clarity of the manuscript.

In line 117, the authors stated that "Seven days from infection animals met the euthanasia criteria". It is not clear why seven days were chosen as the end point of the experiments. Are there any references? The authors should provide some justification. Also, what are the euthanasia criteria? Could the authors give an explanation?

In line 171, the authors stated that "For each marker, images of each area were taken in triplicate in a total of 3 mice (N=3) per group (non-infected controls vs SARS-CoV-2 infected)". It is not clear from this statement how many mice are used in total for the entire set of experiments. It is also not clear whether all or just a portion of the mice infected with SARS-CoV-2 showed viral infections in the central projection neurons as well as a widespread microgliosis.

In Fig. 5, Fig. 6 and Fig. 7, SARS-CoV-2 should be used as in the other figures, since SARS, which is not the proper name for the virus.

Reviewer 2:

Summary:

This manuscript used a mouse model of nasal infection with SARS-CoV-2 to characterize cells infected by virus at 7 days, in the olfactory epithelium and brain. Data are from immunohistochemistry. Histology and staining are good quality; quantification and statistical comparisons are provided. Methods provide good detail. Writing is clear.

The authors conclude that their work provides definitive evidence for SARS-CoV-2 brain neuronal infection and that this may explain aspects of long-Covid in humans. This conclusion is over-stated, since (1) the mouse model used here differs markedly from human (in that the hACE2 receptor is widely driven via the KRT18 promoter, causing broad expression in many cell types that do not strongly express ACE2 in humans); (2) prior work with this mouse model by Perlman found severe blood brain barrier disruption and encephalitis at moderate to higher viral doses, which does not occur in humans (Zheng et al, Nature 2020); and (3) this result contradicts meticulous human post-mortem findings that failed to detect virus in the olfactory bulbs of humans with fatal COVID19 at multiple time points (Khan et al, Cell 2021). In fact, the current work only provides evidence that virus might enter the brain in a transgenic mouse model that differs in key aspects from human. Any conclusions using these findings to consider mechanisms driving long-Covid clinical problems, such as lasting hyposmia, are highly speculative and are not supported.

Specific comments:

Introduction statement claiming that the virus "easily spreads to the brain" (lines 59-60) is not widely accepted or well supported; the 2 references are a low-impact review paper and one of the co-author's reviews. This would need to be stated as speculative, with some evidence supporting this possibility and a wealth of evidence suggesting virus does not widely infect the CNS (with plenty of high-quality references available).

Line 47: there are no microglia in the nose, the cells observed on the nasal septum would be macrophages.

Line 87: This sentence is vague; meaning is not clear. Does this mean to say smell loss is likely due to central pathology rather than peripheral? If so, one would need lots of references to support this claim, if these exist.

All of the results or discussion on mechanisms driving olfactory pathobiology should have very clear distinction stating whether evidence comes from human or animal models, and if animal which specific ones. This is not trivial, since there are major differences in transgenic mice, versus hamster, or human. Also, distinction between post-mortem human experiments versus data or samples from live human subjects is critical. When discussing human data, many (most) studies lack objective psychophysical test data on the subject's olfactory function and some studies do specifically provide these data, so this should be carefully considered as well. Otherwise, broad generalizations as provided are misleading and confusing.

It appears from Methods that all histology was done 7 days post-infection. Why was this time chosen? Since the vast majority of humans with Covid19 hyposmia recover function rapidly (days) and lack any signs or symptoms of actual encephalitis, wouldn't it be logical to assess for CNS virus after a much longer recovery time, i.e. to investigate potential mechanisms underlying lasting hyposmia?

Can you provide any information on why this delivery volume and virus concentration was chosen, and whether any virus-treated mice died, and if so how many?

Line 94: Actually, this mouse model has several caveats that are not discussed, such as the very widespread ACE2 expression differing what is observed in human. Prior reports using this mouse showed high rates of encephalitis, not seen clinically in human. None of this is discussed, key references are ignored.

Sample sizes should be provided in results sections and in figure legends. It appears from methods most of these descriptive results were from n=3 animals?

Lines 232-249: these results (that virus infects sustentacular cells, not neurons) are not novel. This replicates many published studies, and those should be referenced here.

Line 250-257. Are Reelin+ bulb cells Ace2+/Tmprss2+? What do published data sets show in terms of viral entry gene expression in the bulb?

Is there any evidence that protein immunoreactivity identified in brain reflects actual live virus? Are viral RNAs identifiable?

Lines 277-303 are not results. This appears to all be discussion and should be moved, or removed.

Comments on septum versus turbinate infection and inflammation need more clarification. How do viral deliveries in this paper versus other reports differ? For instance, it appears you used a 50 ul instillation to the naris under isofluorane. Others have used much smaller volumes with repeated droplets oer some time interval and differing viral concentration. Might this impact delivery to lateral nasal cavity versus medial areas? One might naively expect nearly all of a large volume delivery to a mouse actively breathing thru the nose

to be swallowed and/or aspirated.

Line 357: It is stated that mechanisms driving smell loss with COVID19 "remain speculative". Yet, the work from Lomvardas lab (in Cell) provides strong evidence for mechanisms driving acute olfactory loss with COVID, as does work from Khan et al from Mombaerts (in Cell); and work from Finlay et al (in Sci Transl Med) provides evidence for mechanisms driving long-COVID hyposmia. None of this is discussed.

Line 384: Are the macrophages actually infected, or have they phagocytosed immunoreactive protein from other infected cells? Is this a possibility? A lack of nearby viral-labeled epithelial cells does not fully exclude this possibility, as they could now be removed or could be transient and/or present on nearby sections.

The Khan et al postmortem study is not discussed. If brain infection is seen at 7 days in this mouse model, yet Khan et al could never find an infected neuron in the bulb of post mortem humans at a variety of time points post infection, looking at a large sample size and probing for multiple viral proteins and for viral RNAs (all of which labeled sus cells in the nose), how do you reconcile these results? The simplest answer is that the results reported here occur only in this transgenic animal model but not in humans; or possibly (yet unlikely) that any transient infection in brain of humans must be rapidly and very efficiently and completely cleared (by a remarkable mechanism that has not been described).

## References

[B1] Abe M, Oshima RG (1990) A single human keratin 18 gene is expressed in diverse epithelial cells of transgenic mice. J Cell Biol 111:1197–1206. 10.1083/jcb.111.3.1197 1697294 PMC2116298

[B2] Awogbindin IO, Ben-Azu B, Olusola BA, Akinluyi ET, Adeniyi PA, Di Paolo T, Tremblay ME (2021) Microglial implications in SARS-CoV-2 infection and COVID-19: lessons from viral RNA neurotropism and possible relevance to Parkinson's disease. Front Cell Neurosci 15:670298. 10.3389/fncel.2021.670298 34211370 PMC8240959

[B3] Beckman D, et al. (2022) SARS-CoV-2 infects neurons and induces neuroinflammation in a non-human primate model of COVID-19. Cell Rep 41:111573. 10.1016/j.celrep.2022.111573 36288725 PMC9554328

[B4] Bergmann CC, Silverman RH (2020) COVID-19: coronavirus replication, pathogenesis, and therapeutic strategies. Cleve Clin J Med 87:321–327. 10.3949/ccjm.87a.20047 32366502

[B5] Bilinska K, Butowt R (2020) Anosmia in COVID-19: a bumpy road to establishing a cellular mechanism. ACS Chem Neurosci 11:2152–2155. 10.1021/acschemneuro.0c00406 32673476 PMC7467568

[B6] Bilinska K, Jakubowska P, Von Bartheld CS, Butowt R (2020) Expression of the SARS-CoV-2 entry proteins, ACE2 and TMPRSS2, in cells of the olfactory epithelium: identification of cell types and trends with age. ACS Chem Neurosci 11:1555–1562. 10.1021/acschemneuro.0c00210 32379417 PMC7241737

[B7] Blackbeard J, O'Dea KP, Wallace VC, Segerdahl A, Pheby T, Takata M, Field MJ, Rice AS (2007) Quantification of the rat spinal microglial response to peripheral nerve injury as revealed by immunohistochemical image analysis and flow cytometry. J Neurosci Methods 164:207–217. 10.1016/j.jneumeth.2007.04.013 17553569 PMC2726922

[B8] Blume C, et al. (2021) A novel ACE2 isoform is expressed in human respiratory epithelia and is upregulated in response to interferons and RNA respiratory virus infection. Nat Genet 53:205–214. 10.1038/s41588-020-00759-x 33432184

[B9] Borst K, Dumas AA, Prinz M (2021) Microglia: immune and non-immune functions. Immunity 54:2194–2208. 10.1016/j.immuni.2021.09.014 34644556

[B10] Bourgon C, Albin AS, Ando-Grard O, Da Costa B, Domain R, Korkmaz B, Klonjkowski B, Le Poder S, Meunier N (2022) Neutrophils play a major role in the destruction of the olfactory epithelium during SARS-CoV-2 infection in hamsters. Cell Mol Life Sci 79:616. 10.1007/s00018-022-04643-1 36460750 PMC9734468

[B11] Brann DH, et al. (2020) Non-neuronal expression of SARS-CoV-2 entry genes in the olfactory system suggests mechanisms underlying COVID-19-associated anosmia. Sci Adv 6:eabc5801. 10.1126/sciadv.abc5801 32937591 PMC10715684

[B12] Brodin P (2021) Immune determinants of COVID-19 disease presentation and severity. Nat Med 27:28–33. 10.1038/s41591-020-01202-8 33442016

[B13] Bryche B, et al. (2020) Massive transient damage of the olfactory epithelium associated with infection of sustentacular cells by SARS-CoV-2 in golden Syrian hamsters. Brain Behav Immun 89:579–586. 10.1016/j.bbi.2020.06.032 32629042 PMC7332942

[B14] Burks SM, Rosas-Hernandez H, Alejandro Ramirez-Lee M, Cuevas E, Talpos JC (2021) Can SARS-CoV-2 infect the central nervous system via the olfactory bulb or the blood-brain barrier? Brain Behav Immun 95:7–14. 10.1016/j.bbi.2020.12.031 33412255 PMC7836942

[B15] Butowt R, Bilinska K, von Bartheld CS (2023) Olfactory dysfunction in COVID-19: new insights into the underlying mechanisms. Trends Neurosci 46:75–90. 10.1016/j.tins.2022.11.003 36470705 PMC9666374

[B16] Butowt R, Meunier N, Bryche B, von Bartheld CS (2021) The olfactory nerve is not a likely route to brain infection in COVID-19: a critical review of data from humans and animal models. Acta Neuropathol 141:809–822. 10.1007/s00401-021-02314-2 33903954 PMC8075028

[B17] Calvier L, Drelich A, Hsu J, Tseng CT, Mina Y, Nath A, Kounnas MZ, Herz J (2023) Circulating Reelin promotes inflammation and modulates disease activity in acute and long COVID-19 cases. Front Immunol 14:1185748. 10.3389/fimmu.2023.1185748 37441066 PMC10333573

[B18] Cantuti-Castelvetri L, et al. (2020) Neuropilin-1 facilitates SARS-CoV-2 cell entry and infectivity. Science 370:856–860. 10.1126/science.abd2985 33082293 PMC7857391

[B19] Cappelletti G, et al. (2023) Human motor neurons derived from induced pluripotent stem cells are susceptible to SARS-CoV-2 infection. Front Cell Neurosci 17:1285836. 10.3389/fncel.2023.1285836 38116398 PMC10728732

[B20] Carossino M, et al. (2022) Fatal neurodissemination and SARS-CoV-2 tropism in K18-hACE2 mice is only partially dependent on hACE2 expression. Viruses 14:535. 10.3390/v14030535 35336942 PMC8955233

[B21] Choi S, et al. (2024) A longitudinal molecular and cellular lung atlas of lethal SARS-CoV-2 infection in K18-hACE2 transgenic mice. EBioMedicine 99:104932. 10.1016/j.ebiom.2023.104932 38118400 PMC10772566

[B22] Choi R, Gupta R, Finlay JB, Goldstein BJ (2022) Olfactory dysfunction and COVID-19. Oper Tech Otolayngol Head Neck Surg 33:141–146. 10.1016/j.otot.2022.04.010 35505955 PMC9050605

[B23] Cooper KW, et al. (2020) COVID-19 and the chemical senses: supporting players take center stage. Neuron 107:219–233. 10.1016/j.neuron.2020.06.032 32640192 PMC7328585

[B24] Dedoni S, Avdoshina V, Camoglio C, Siddi C, Fratta W, Scherma M, Fadda P (2022) K18- and CAG-hACE2 transgenic mouse models and SARS-CoV-2: implications for neurodegeneration research. Molecules 27:4142. 10.3390/molecules27134142 35807384 PMC9268291

[B25] de Melo GD, et al. (2021) COVID-19-related anosmia is associated with viral persistence and inflammation in human olfactory epithelium and brain infection in hamsters. Sci Transl Med 13:eabf8396. 10.1126/scitranslmed.abf8396 33941622 PMC8158965

[B26] Dey R, Bishayi B (2023) Microglial inflammatory responses to SARS-CoV-2 infection: a comprehensive review. Cell Mol Neurobiol 44:2. 10.1007/s10571-023-01444-3 38099973 PMC11407175

[B27] Domingues RB, et al. (2020) First case of SARS-COV-2 sequencing in cerebrospinal fluid of a patient with suspected demyelinating disease. J Neurol 267:3154–3156. 10.1007/s00415-020-09996-w 32564153 PMC7305694

[B28] Dong W, et al. (2022) The K18-human ACE2 transgenic mouse model recapitulates non-severe and severe COVID-19 in response to an infectious dose of the SARS-CoV-2 virus. J Virol 96:e0096421. 10.1128/JVI.00964-21 34668775 PMC8754221

[B29] Dusedau HP, et al. (2021) Influenza A virus (H1N1) infection induces microglial activation and temporal dysbalance in glutamatergic synaptic transmission. mBio 12:e0177621. 10.1128/mBio.01776-21 34700379 PMC8546584

[B30] Eberhardt N, et al. (2023) SARS-CoV-2 infection triggers pro-atherogenic inflammatory responses in human coronary vessels. Nat Cardiovasc Res 2:899–916. 10.1038/s44161-023-00336-5 38076343 PMC10702930

[B31] Esposito F, Cirillo M, De Micco R, Caiazzo G, Siciliano M, Russo AG, Monari C, Coppola N, Tedeschi G, Tessitore A (2022) Olfactory loss and brain connectivity after COVID-19. Hum Brain Mapp 43:1548–1560. 10.1002/hbm.25741 35083823 PMC8886650

[B32] Esposito F, Cirillo M, De Micco R, Caiazzo G, Siciliano M, Russo AG, Monari C, Coppola N, Tedeschi G, Tessitore A (2023) Olfactory loss and brain connectivity after COVID-19: structural follow-up at one year. Neural Plast 2023:6496539. 10.1155/2023/6496539 37159825 PMC10163964

[B33] Farbman AI, Margolis FL (1980) Olfactory marker protein during ontogeny: immunohistochemical localization. Dev Biol 74:205–215. 10.1016/0012-1606(80)90062-7 7350009

[B34] Fernandez-Castaneda A, et al. (2022) Mild respiratory COVID can cause multi-lineage neural cell and myelin dysregulation. Cell 185:2452–2468.e16. 10.1016/j.cell.2022.06.008 35768006 PMC9189143

[B35] Finlay JB, et al. (2022) Persistent post-COVID-19 smell loss is associated with immune cell infiltration and altered gene expression in olfactory epithelium. Sci Transl Med 14:eadd0484. 10.1126/scitranslmed.add0484 36542694 PMC10317309

[B36] Fodoulian L, et al. (2020) SARS-CoV-2 receptors and entry genes are expressed in the human olfactory neuroepithelium and brain. iScience 23:101839. 10.1016/j.isci.2020.101839 33251489 PMC7685946

[B37] Frere JJ, et al. (2022) SARS-CoV-2 infection in hamsters and humans results in lasting and unique systemic perturbations after recovery. Sci Transl Med 14:eabq3059. 10.1126/scitranslmed.abq3059 35857629 PMC9210449

[B38] Garg RK, Paliwal VK, Gupta A (2021) Encephalopathy in patients with COVID-19: a review. J Med Virol 93:206–222. 10.1002/jmv.26207 32558956

[B39] Golden JW, et al. (2020) Human angiotensin-converting enzyme 2 transgenic mice infected with SARS-CoV-2 develop severe and fatal respiratory disease. JCI Insight 5:e142032. 10.1172/jci.insight.142032 32841215 PMC7566707

[B40] Golden JW, et al. (2022) Hamsters expressing human angiotensin-converting enzyme 2 develop severe disease following exposure to SARS-CoV-2. mBio 13:e0290621. 10.1128/mbio.02906-21 35073750 PMC8787465

[B41] Guo Z, Packard A, Krolewski RC, Harris MT, Manglapus GL, Schwob JE (2010) Expression of pax6 and sox2 in adult olfactory epithelium. J Comp Neurol 518:4395–4418. 10.1002/cne.22463 20852734 PMC2940252

[B42] Harkema JR, Carey SA, Wagner JG (2006) The nose revisited: a brief review of the comparative structure, function, and toxicologic pathology of the nasal epithelium. Toxicol Pathol 34:252–269. 10.1080/01926230600713475 16698724

[B43] Hoogland IC, Houbolt C, van Westerloo DJ, van Gool WA, van de Beek D (2015) Systemic inflammation and microglial activation: systematic review of animal experiments. J Neuroinflammation 12:114. 10.1186/s12974-015-0332-6 26048578 PMC4470063

[B44] Huang C, et al. (2020) Clinical features of patients infected with 2019 novel coronavirus in Wuhan, China. Lancet 395:497–506. 10.1016/S0140-6736(20)30183-5 31986264 PMC7159299

[B45] Iadecola C, Anrather J, Kamel H (2020) Effects of COVID-19 on the nervous system. Cell 183:16–27.e11. 10.1016/j.cell.2020.08.028 32882182 PMC7437501

[B46] Jeong GU, et al. (2022) SARS-CoV-2 infection of microglia elicits proinflammatory activation and apoptotic cell death. Microbiol Spectr 10:e0109122. 10.1128/spectrum.01091-22 35510852 PMC9241873

[B47] Kaufer C, et al. (2022) Microgliosis and neuronal proteinopathy in brain persist beyond viral clearance in SARS-CoV-2 hamster model. EBioMedicine 79:103999. 10.1016/j.ebiom.2022.103999 35439679 PMC9013202

[B48] Khan M, et al. (2021) Visualizing in deceased COVID-19 patients how SARS-CoV-2 attacks the respiratory and olfactory mucosae but spares the olfactory bulb. Cell 184:5932–5949.e15. 10.1016/j.cell.2021.10.027 34798069 PMC8564600

[B49] Khan M, et al. (2022) Anatomical barriers against SARS-CoV-2 neuroinvasion at vulnerable interfaces visualized in deceased COVID-19 patients. Neuron 110:3919–3935.e16. 10.1016/j.neuron.2022.11.007 36446381 PMC9647025

[B50] Kishimoto-Urata M, et al. (2022) Prolonged and extended impacts of SARS-CoV-2 on the olfactory neurocircuit. Sci Rep 12:5728. 10.1038/s41598-022-09731-7 35388072 PMC8987081

[B51] Krasemann S, et al. (2017) The TREM2-APOE pathway drives the transcriptional phenotype of dysfunctional microglia in neurodegenerative diseases. Immunity 47:566–581.e9. 10.1016/j.immuni.2017.08.008 28930663 PMC5719893

[B52] Kumari P, Rothan HA, Natekar JP, Stone S, Pathak H, Strate PG, Arora K, Brinton MA, Kumar M (2021) Neuroinvasion and encephalitis following intranasal inoculation of SARS-CoV-2 in K18-hACE2 mice. Viruses 13:132. 10.3390/v13010132 33477869 PMC7832889

[B53] Lamers MM, Haagmans BL (2022) SARS-CoV-2 pathogenesis. Nat Rev Microbiol 20:270–284. 10.1038/s41579-022-00713-0 35354968

[B54] Lappe-Siefke C, Goebbels S, Gravel M, Nicksch E, Lee J, Braun PE, Griffiths IR, Nave KA (2003) Disruption of Cnp1 uncouples oligodendroglial functions in axonal support and myelination. Nat Genet 33:366–374. 10.1038/ng1095 12590258

[B55] Lechien JR, et al. (2020) Olfactory and gustatory dysfunctions as a clinical presentation of mild-to-moderate forms of the coronavirus disease (COVID-19): a multicenter European study. Eur Arch Otorhinolaryngol 277:2251–2261. 10.1007/s00405-020-05965-1 32253535 PMC7134551

[B56] Lee IT, et al. (2020) ACE2 localizes to the respiratory cilia and is not increased by ACE inhibitors or ARBs. Nat Commun 11:5453. 10.1038/s41467-020-19145-6 33116139 PMC7595232

[B57] Li YC, Bai WZ, Hashikawa T (2020) The neuroinvasive potential of SARS-CoV2 may play a role in the respiratory failure of COVID-19 patients. J Med Virol 92:552–555. 10.1002/jmv.25728 32104915 PMC7228394

[B58] Lier J, Streit WJ, Bechmann I (2021) Beyond activation: characterizing microglial functional phenotypes. Cells 10:2236. 10.3390/cells10092236 34571885 PMC8464670

[B59] Luczo JM, Edwards SJ, Ardipradja K, Suen WW, Au GG, Marsh GA, Godde N, Rootes CL, Bingham J, Sundaramoorthy V (2024) SARS-CoV and SARS-CoV-2 display limited neuronal infection and lack the ability to transmit within synaptically connected axons in stem cell-derived human neurons. J Neurovirol 30:39–51. 10.1007/s13365-023-01187-3 38172412 PMC11035468

[B60] Lyoo KS, et al. (2022) Direct neuronal infection of SARS-CoV-2 reveals cellular and molecular pathology of chemosensory impairment of COVID-19 patients. Emerg Microbes Infect 11:406–411. 10.1080/22221751.2021.2024095 34962444 PMC8803065

[B61] Mao L, et al. (2020) Neurologic manifestations of hospitalized patients with coronavirus disease 2019 in Wuhan, China. JAMA Neurol 77:683–690. 10.1001/jamaneurol.2020.1127 32275288 PMC7149362

[B62] Mao T, et al. (2022) Unadjuvanted intranasal spike vaccine elicits protective mucosal immunity against sarbecoviruses. Science 378:eabo2523. 10.1126/science.abo2523 36302057 PMC9798903

[B63] Martin-Lopez E, Blanchart A, De Carlos JA, Lopez-Mascaraque L (2011) Dab1 (Disable homolog-1) reelin adaptor protein is overexpressed in the olfactory bulb at early postnatal stages. PLoS One 6:e26673. 10.1371/journal.pone.0026673 22046330 PMC3201967

[B64] Martin-Lopez E, Ishiguro K, Greer CA (2019a) The laminar organization of piriform cortex follows a selective developmental and migratory program established by cell lineage. Cereb Cortex 29:1–16. 10.1093/cercor/bhx291 29136113 PMC7199997

[B65] Martin-Lopez E, Xu C, Liberia T, Meller SJ, Greer CA (2019b) Embryonic and postnatal development of mouse olfactory tubercle. Mol Cell Neurosci 98:82–96. 10.1016/j.mcn.2019.06.002 31200100 PMC11993912

[B66] Matschke J, et al. (2020) Neuropathology of patients with COVID-19 in Germany: a post-mortem case series. Lancet Neurol 19:919–929. 10.1016/S1474-4422(20)30308-2 33031735 PMC7535629

[B67] Maurya DK, Henriques T, Marini M, Pedemonte N, Galietta LJ, Rock JR, Harfe BD, Menini A (2015) Development of the olfactory epithelium and nasal glands in TMEM16A-/- and TMEM16A+/+ mice. PLoS One 10:e0129171. 10.1371/journal.pone.0129171 26067252 PMC4465891

[B68] McCray PB Jr, et al. (2007) Lethal infection of K18-hACE2 mice infected with severe acute respiratory syndrome coronavirus. J Virol 81:813–821. 10.1128/JVI.02012-06 17079315 PMC1797474

[B69] McWhorter FY, Wang T, Nguyen P, Chung T, Liu WF (2013) Modulation of macrophage phenotype by cell shape. Proc Natl Acad Sci U S A 110:17253–17258. 10.1073/pnas.1308887110 24101477 PMC3808615

[B70] Meinhardt J, et al. (2021) Olfactory transmucosal SARS-CoV-2 invasion as a port of central nervous system entry in individuals with COVID-19. Nat Neurosci 24:168–175. 10.1038/s41593-020-00758-5 33257876

[B71] Meinhardt J, Streit S, Dittmayer C, Manitius RV, Radbruch H, Heppner FL (2024) The neurobiology of SARS-CoV-2 infection. Nat Rev Neurosci 25:30–42. 10.1038/s41583-023-00769-8 38049610

[B72] Meller SJ, Hernandez L, Martin-Lopez E, Kloos ZA, Liberia T, Greer CA (2023) Microglia maintain homeostatic conditions in the developing rostral migratory stream. eNeuro 10:ENEURO.0197-22.2023. 10.1523/ENEURO.0197-22.2023 PMC9910579 36697258

[B73] Merad M, Blish CA, Sallusto F, Iwasaki A (2022) The immunology and immunopathology of COVID-19. Science 375:1122–1127. 10.1126/science.abm8108 35271343 PMC12828912

[B74] Monje M, Iwasaki A (2022) The neurobiology of long COVID. Neuron 110:3484–3496. 10.1016/j.neuron.2022.10.006 36288726 PMC9537254

[B75] Moriguchi T, et al. (2020) A first case of meningitis/encephalitis associated with SARS-coronavirus-2. Int J Infect Dis 94:55–58. 10.1016/j.ijid.2020.03.062 32251791 PMC7195378

[B76] Neville KR, Haberly LB (2004) Olfactory cortex. In: The synaptic organization of the brain, Ed 5 (Shepherd GM, ed), pp 67. USA: Oxford University Press.

[B77] Oladunni FS, et al. (2020) Lethality of SARS-CoV-2 infection in K18 human angiotensin-converting enzyme 2 transgenic mice. Nat Commun 11:6122. 10.1038/s41467-020-19891-7 33257679 PMC7705712

[B78] Olivarria GM, et al. (2022) Microglia do not restrict SARS-CoV-2 replication following infection of the central nervous system of K18-human ACE2 transgenic mice. J Virol 96:e0196921. 10.1128/jvi.01969-21 34935438 PMC8865461

[B79] Paniz-Mondolfi A, Bryce C, Grimes Z, Gordon RE, Reidy J, Lednicky J, Sordillo EM, Fowkes M (2020) Central nervous system involvement by severe acute respiratory syndrome coronavirus-2 (SARS-CoV-2). J Med Virol 92:699–702. 10.1002/jmv.25915 32314810 PMC7264598

[B80] Pellegrini L, Albecka A, Mallery DL, Kellner MJ, Paul D, Carter AP, James LC, Lancaster MA (2020) SARS-CoV-2 infects the brain choroid plexus and disrupts the blood-CSF barrier in human brain organoids. Cell Stem Cell 27:951–961.e5. 10.1016/j.stem.2020.10.001 33113348 PMC7553118

[B81] Poloni TE, et al. (2021) COVID-19-related neuropathology and microglial activation in elderly with and without dementia. Brain Pathol 31:e12997. 10.1111/bpa.12997 34145669 PMC8412067

[B82] Priyal, Sehgal V, Kapila S, Taneja R, Mehmi P, Gulati N (2023) Review of neurological manifestations of SARS-CoV-2. Cureus 15:e38194. 10.7759/cureus.38194 37257164 PMC10223874

[B83] Ramani A, et al. (2020) SARS-CoV-2 targets neurons of 3D human brain organoids. EMBO J 39:e106230. 10.15252/embj.2020106230 32876341 PMC7560208

[B84] Savelieff MG, Feldman EL, Stino AM (2022) Neurological sequela and disruption of neuron-glia homeostasis in SARS-CoV-2 infection. Neurobiol Dis 168:105715. 10.1016/j.nbd.2022.105715 35364273 PMC8963977

[B85] Seehusen F, et al. (2022) Neuroinvasion and neurotropism by SARS-CoV-2 variants in the K18-hACE2 mouse. Viruses 14:1020. 10.3390/v14051020 35632761 PMC9146514

[B86] Shepherd GM, Rowe TB, Greer CA (2021) An evolutionary microcircuit approach to the neural basis of high dimensional sensory processing in olfaction. Front Cell Neurosci 15:658480. 10.3389/fncel.2021.658480 33994949 PMC8120314

[B87] Shimizu S, et al. (2024) SARS-CoV-2 induces inflammation and intracranial infection through the olfactory epithelium-olfactory bulb pathway in non-human primates. J Neuroimmunol 387:578288. 10.1016/j.jneuroim.2024.578288 38237527

[B88] Sia SF, et al. (2020) Pathogenesis and transmission of SARS-CoV-2 in golden hamsters. Nature 583:834–838. 10.1038/s41586-020-2342-5 32408338 PMC7394720

[B89] Soltys Z, Ziaja M, Pawlinski R, Setkowicz Z, Janeczko K (2001) Morphology of reactive microglia in the injured cerebral cortex. Fractal analysis and complementary quantitative methods. J Neurosci Res 63:90–97. 10.1002/1097-4547(20010101)63:1<90::AID-JNR11>3.0.CO;2-9 11169618

[B90] Song E, et al. (2021) Neuroinvasion of SARS-CoV-2 in human and mouse brain. J Exp Med 218:e20202135. 10.1084/jem.20202135 33433624 PMC7808299

[B91] Stein SR, et al. (2022) SARS-CoV-2 infection and persistence in the human body and brain at autopsy. Nature 612:758–763. 10.1038/s41586-022-05542-y 36517603 PMC9749650

[B92] Torabi A, et al. (2020) Proinflammatory cytokines in the olfactory mucosa result in COVID-19 induced anosmia. ACS Chem Neurosci 11:1909–1913. 10.1021/acschemneuro.0c00249 32525657

[B93] Tsukahara T, Brann DH, Datta SR (2023) Mechanisms of SARS-CoV-2-associated anosmia. Physiol Rev 103:2759–2766. 10.1152/physrev.00012.2023 37342077 PMC10625840

[B94] Ueha R, Ito T, Furukawa R, Kitabatake M, Ouji-Sageshima N, Ueha S, Koyama M, Uranaka T, Kondo K, Yamasoba T (2022) Oral SARS-CoV-2 inoculation causes nasal viral infection leading to olfactory bulb infection: an experimental study. Front Cell Infect Microbiol 12:924725. 10.3389/fcimb.2022.924725 35770069 PMC9234459

[B95] Ueha R, Kondo K, Kagoya R, Shichino S, Shichino S, Yamasoba T (2021) ACE2, TMPRSS2, and Furin expression in the nose and olfactory bulb in mice and humans. Rhinology 59:105–109. 10.4193/Rhin20.324 33249429

[B96] Urata S, Maruyama J, Kishimoto-Urata M, Sattler RA, Cook R, Lin N, Yamasoba T, Makishima T, Paessler S (2021) Regeneration profiles of olfactory epithelium after SARS-CoV-2 infection in golden Syrian hamsters. ACS Chem Neurosci 12:589–595. 10.1021/acschemneuro.0c00649 33522795 PMC7874468

[B97] Varatharaj A, et al. (2020) Neurological and neuropsychiatric complications of COVID-19 in 153 patients: a UK-wide surveillance study. Lancet Psychiatry 7:875–882. 10.1016/S2215-0366(20)30287-X 32593341 PMC7316461

[B98] Vassar R, Ngai J, Axel R (1993) Spatial segregation of odorant receptor expression in the mammalian olfactory epithelium. Cell 74:309–318. 10.1016/0092-8674(93)90422-M 8343958

[B99] Verma AK, Zheng J, Meyerholz DK, Perlman S (2022) SARS-CoV-2 infection of sustentacular cells disrupts olfactory signaling pathways. JCI Insight 7:e160277. 10.1172/jci.insight.160277 36378534 PMC9869979

[B100] Vidal E, et al. (2022) Chronological brain lesions after SARS-CoV-2 infection in hACE2-transgenic mice. Vet Pathol 59:613–626. 10.1177/03009858211066841 34955064 PMC9207990

[B101] Villadiego J, et al. (2023) Full protection from SARS-CoV-2 brain infection and damage in susceptible transgenic mice conferred by MVA-CoV2-S vaccine candidate. Nat Neurosci 26:226–238. 10.1038/s41593-022-01242-y 36624276

[B102] Wesson DW (2020) The tubular striatum. J Neurosci 40:7379–7386. 10.1523/JNEUROSCI.1109-20.2020 32968026 PMC7511186

[B103] Yang L, et al. (2024) SARS-CoV-2 infection causes dopaminergic neuron senescence. Cell Stem Cell 31:196–211.e6. 10.1016/j.stem.2023.12.012 38237586 PMC10843182

[B104] Ye Q, et al. (2021) SARS-CoV-2 infection in the mouse olfactory system. Cell Discov 7:49. 10.1038/s41421-021-00290-1 34230457 PMC8260584

[B105] Yinda CK, et al. (2021) K18-hACE2 mice develop respiratory disease resembling severe COVID-19. PLoS Pathog 17:e1009195. 10.1371/journal.ppat.1009195 33465158 PMC7875348

[B106] Yu P, Deng W, Bao L, Qu Y, Xu Y, Zhao W, Han Y, Qin C (2022) Comparative pathology of the nasal epithelium in K18-hACE2 Tg mice, hACE2 Tg mice, and hamsters infected with SARS-CoV-2. Vet Pathol 59:602–612. 10.1177/03009858211071016 35094625 PMC9208069

[B107] Zabel MK, Kirsch WM (2013) From development to dysfunction: microglia and the complement cascade in CNS homeostasis. Ageing Res Rev 12:749–756. 10.1016/j.arr.2013.02.001 23419464 PMC3700678

[B108] Zapiec B, Mombaerts P (2020) The zonal organization of odorant receptor gene choice in the main olfactory epithelium of the mouse. Cell Rep 30:4220–4234.e25. 10.1016/j.celrep.2020.02.110 32209480

[B109] Zazhytska M, et al. (2022) Non-cell-autonomous disruption of nuclear architecture as a potential cause of COVID-19-induced anosmia. Cell 185:1052–1064.e12. 10.1016/j.cell.2022.01.024 35180380 PMC8808699

[B110] Zhao X, Gao F (2024) Novel omicron variants enhance anchored recognition of TMEM106B: a new pathway for SARS-CoV-2 cellular invasion. J Phys Chem Lett 15:671–680. 10.1021/acs.jpclett.3c03296 38206837

[B111] Zheng J, et al. (2021) COVID-19 treatments and pathogenesis including anosmia in K18-hACE2 mice. Nature 589:603–607. 10.1038/s41586-020-2943-z 33166988 PMC7855185

[B112] Ziuzia-Januszewska L, Januszewski M (2022) Pathogenesis of olfactory disorders in COVID-19. Brain Sci 12:449. 10.3390/brainsci12040449 35447981 PMC9029941

